# Enhancing auxin accumulation in maize root tips improves root growth and dwarfs plant height

**DOI:** 10.1111/pbi.12751

**Published:** 2017-06-23

**Authors:** Zhaoxia Li, Xinrui Zhang, Yajie Zhao, Yujie Li, Guangfeng Zhang, Zhenghua Peng, Juren Zhang

**Affiliations:** ^1^ School of Life Science Shandong University Jinan Shandong China

**Keywords:** maize, transgenic breeding, ideal plant type, root architecture modification, yield

## Abstract

Maize is a globally important food, feed crop and raw material for the food and energy industry. Plant architecture optimization plays important roles in maize yield improvement. PIN‐FORMED (PIN) proteins are important for regulating auxin spatiotemporal asymmetric distribution in multiple plant developmental processes. In this study, *ZmPIN1a* overexpression in maize increased the number of lateral roots and inhibited their elongation, forming a developed root system with longer seminal roots and denser lateral roots. *ZmPIN1a* overexpression reduced plant height, internode length and ear height. This modification of the maize phenotype increased the yield under high‐density cultivation conditions, and the developed root system improved plant resistance to drought, lodging and a low‐phosphate environment. IAA concentration, transport capacity determination and application of external IAA indicated that *ZmPIN1a* overexpression led to increased IAA transport from shoot to root. The increase in auxin in the root enabled the plant to allocate more carbohydrates to the roots, enhanced the growth of the root and improved plant resistance to environmental stress. These findings demonstrate that maize plant architecture can be improved by root breeding to create an ideal phenotype for further yield increases.

## Introduction

For more than 100 years, maize has been a major food, feed and industrial feedstock for bioproducts around the world. The combination of population growth and climate change with the degradation and scarcity of natural resources threatens food security and the livelihoods of millions of resource‐poor people. Improving crop yields under optimal and complex abiotic and biotic stress conditions is the most daunting challenge faced by breeders.

Traditional breeding for maize yield has achieved many successes. Trait changes that increase resistance to a wide variety of biotic and abiotic stresses (e.g. drought tolerance) have been the most numerous, but morphological and physiological changes that promote efficiency in growth, development and partitioning (e.g. smaller tassels) have also been recorded (Duvick, 2005; Ribaut *et al*., 2009, 2010; Tester and Langridge, 2010). The plant density that maximizes corn grain and silage yield has increased over time. Plant densities that maximize grain and forage yield are higher than currently recommended plant densities. Adjusting plant density is likely one of the best ways to increase current yield levels. This consideration favours varieties with outstanding resistance to close planting. The ideal phenotype in maize breeding includes high biomass and yield, high canopy photosynthetic potential, good lodging resistance and lower empty stalk rates in high‐density cultivation, high grain‐filling velocity, high thousand‐grain weight, stable performance and biotic and abiotic stress resistance (Araus *et al*., 2008; Lee and Tollenaar, 2007; Ribaut *et al*., 2009). It is difficult to breed this type of maize variety using traditional breeding.

The importance of a deep and vigorous root system for higher yield has recently been recognized in diverse crops (Kell, 2011; Smith and De Smet, 2012). Root system characteristics have not been extensively examined within maize breeding because of their complexity and inaccessibility underground (Lynch, 2015). Auxin plays a central role in many aspects of plant morphogenesis. The well‐characterized auxin‐associated phenotypes in roots are a dose‐dependent increase in the length of epidermis‐derived root hairs, a bimodal effect of auxin concentration on primary root length, a dose‐dependent increase in the number of lateral root primordia and the response to gravity (Ishida *et al*., 2008; Peret *et al*., 2009; Pitts *et al*., 1998). Unique auxin polar transport was found to be critical for forming and maintaining the local auxin concentration. Over the past decades, research on polar auxin transport mutants of *Arabidopsis* has indicated that local auxin concentrations and polar auxin transport are important for root morphology and development (Habets and Offringa, 2014; Overvoorde *et al*., 2010). Polar auxin transport is mediated by three classes of proteins: the AUX1/LAX family, the PIN‐FORMED (PIN) family and P‐glycoprotein ABCB subfamily proteins (Petrasek and Friml, 2009). PINs and PGP‐mediated auxin transport in maize have been verified in relation to cellular differentiation during maize embryogenesis, endosperm development (Forestan and Varotto, 2012; Forestan *et al*., 2010) and branching (Gallavotti *et al*., 2008; Knoller *et al*., 2010; Multani *et al*., 2003). However, the relationships between polar auxin transport, maize plant type and root system architecture are still unclear.

Changing auxin concentrations and signalling to alter or modify maize plant morphology and/or growth is a challenging project for biologists and breeders. In this report, the overexpressed *ZmPIN1a* lines of maize were produced to investigate the effects of auxin concentration alteration in roots on the morphology and development of plants. Overexpression of *ZmPIN1a* acropetally increased auxin transport in roots and formed a root system with longer seminal roots and shorter dense lateral roots, along with dwarfed plant height. *ZmPIN1a* sense lines also showed enhanced maize yields under high‐density planting. To understand the effects of increased auxin concentration in maize roots, RNA‐seq was accomplished to explore the change in transcriptome, and many of the genes involved in auxin signalling or participating in ethylene metabolism and signalling processes showed up‐ or down‐expression, which implied the modification of plant architecture in *ZmPIN1a* sense lines originated from the increased auxin concentration in the root. This study offers a new strategy for the improvement of plant architecture to breed a new germplasm with an ideal maize phenotype by altering the auxin transport capacity in roots.

## Results

### Candidate gene sequence analyses and expression profile

**Figure 1:**
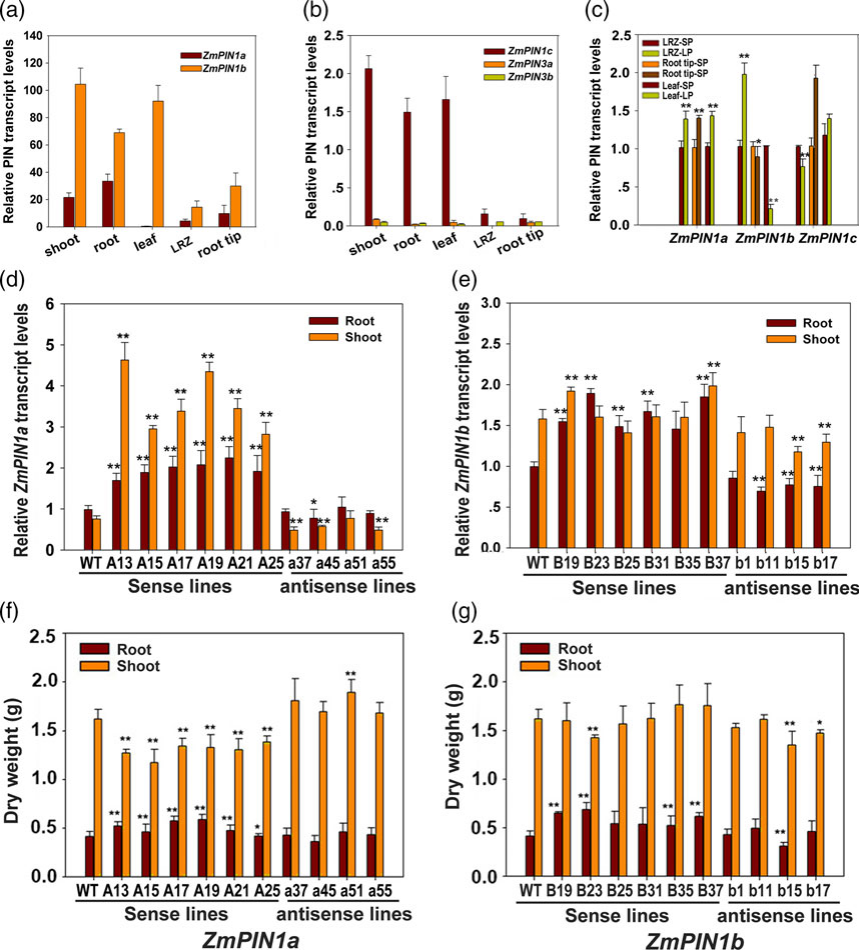
Expression of *ZmPIN*s and selection of transgenic lines with real‐time RT–PCR and biomass measurements. (a), (b) Expression pattern analysis of *PINs* in maize. The root and shoot were cut off from seedlings germinated for eight days, and the leaf, LRZ (1.0–1.5 cm segment containing the lateral root primordia) and root tip were from three‐leaf stage plants. Fold changes in RNA transcripts were calculated using the 2^−ΔΔCt^ method with maize *Actin1* as an internal control: the ∆ values of the target gene and reference gene (maize Actin1) were calculated in the samples, and the values of 1/10 maize Actin1 in the roots were used as the standard to calculate the relative expression levels of the PIN genes. (c) Expression analysis of *ZmPIN1a*,* ZmPIN1b* and *ZmPIN1c* subjected to low‐phosphate starvation (LP). Fold changes in RNA transcripts were calculated using the 2^−ΔΔCt^ method with maize *Actin1* as an internal control, and the values of gene expression levels cultured in normal nutrient solution (SP) were taken as onefold. (d) and (e) Screening *ZmPIN1a* and *ZmPIN1b* transgenic lines for real‐time RT–PCR. (f) and (g) Biomass determination of *ZmPIN1a* and *ZmPIN1b* transgenic lines cultured in vermiculite at the five‐leaf stage. The prefix ‘A’ or ‘B’ in a name denotes the *ZmPIN1a* or *ZmPIN1b* sense line, and an ‘a’ or ‘b’ in a name denotes the *ZmPIN1a* or *ZmPIN1b* antisense line. WT denotes the wild‐type control DH4866. The roots and shoots of eight‐day germinated seedlings were used. The expression levels of genes were analysed using real‐time RT–PCR, and fold changes in RNA transcripts were calculated using the 2^−ΔΔCt^ method with maize *Actin1* as an internal control. The values of the target gene from the roots of the WT line were taken as onefold. The expression levels are expressed as the mean of the relative fold changes from triplicate biological replicates, and the vertical bars represent the standard deviation (*n* = 3). For biomass analysis, the plants in vermiculite were harvested at the five‐leaf stage. DW denotes dry weight. The values are means ± SD (*n* = 10). The asterisks indicate significant differences between transgenic and WT lines at the *0.05 or **0.01 level using the *t*‐test.

Genes orthologous to *AtPIN1* were cloned from maize DH4866 according to the sequence submitted by Carraro *et al*. (2006) using the primers list in Table S1. Sequence analysis showed that four PIN1 orthologous are present in maize, as in rice, and are named ZmPIN1a, ZmPIN1b, ZmPIN1c and ZmPIN1d, which diverged in the large central hydrophilic loop (from amino acid 290~414 of ZmPIN1a). These results are consistent with those of Forestan and Varotto (2012) except for an amino acid substitution in ZmPIN1a (124 Met to Val) from DH4866 compared to B73. As shown in Figure S1a–c, ZmPIN1b and ZmPIN1c share a high degree of similarity with the exception of a few amino acids and are the homologues of OsPIN1a, while ZmPIN1a is the homologue of OsPIN1c. As shown in Figure 1a–c, the six long loop PINs (located in the PM) have distinct expression levels and patterns in maize. Among them, *ZmPIN1b* has the highest expression levels in the upland of maize. *ZmPIN1a* has higher expression levels in the root, and both are Pi‐responsive genes. These observations indicate that these genes may collaboratively contribute to the subtle local auxin distribution in maize plant morphogenesis and participate in root architecture modification under normal and low‐phosphate (LP) conditions.

### Manipulating *ZmPIN1a* and *ZmPIN1b* modified maize root architecture and plant height

**Figure 2:**
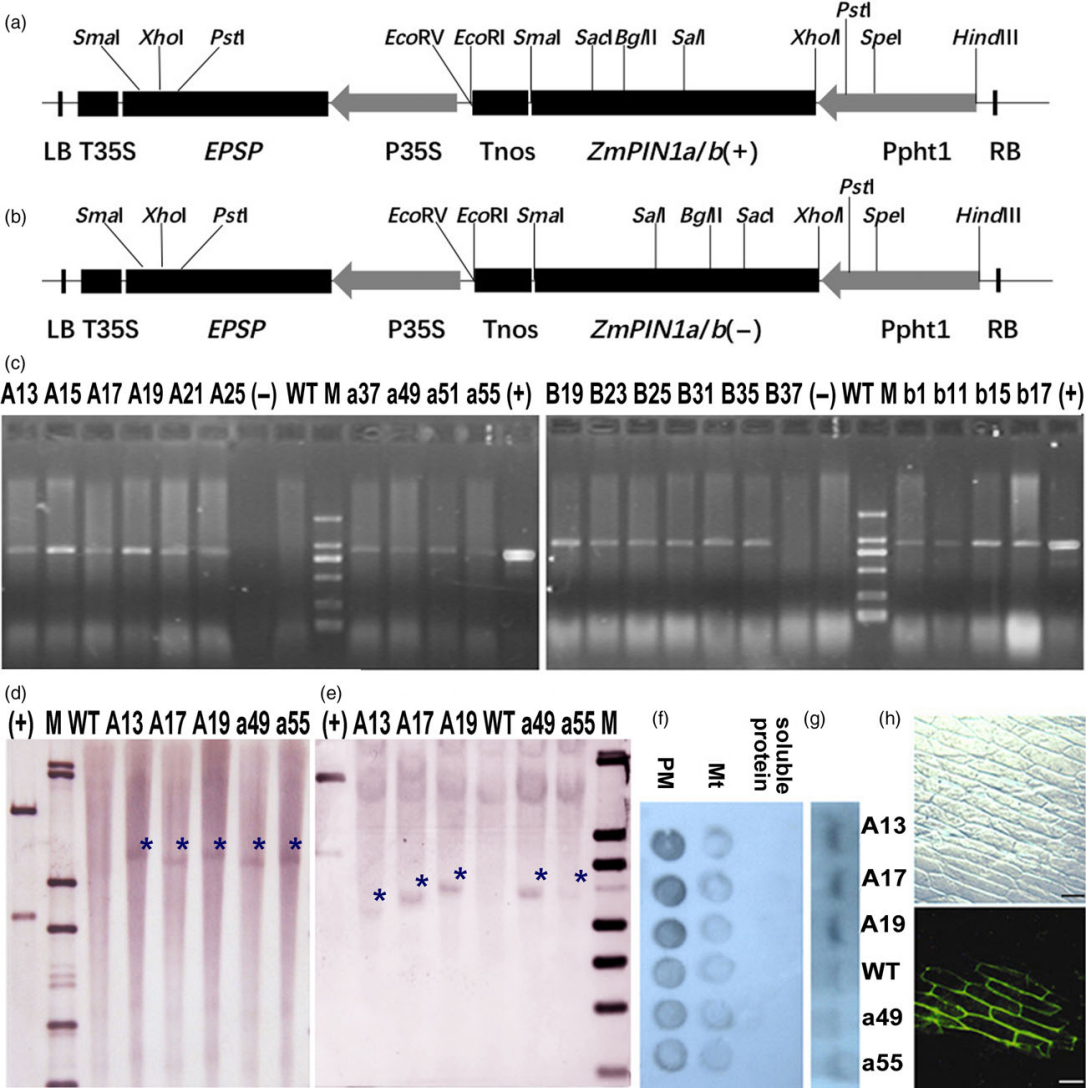
T‐DNA region and molecular identification of transgenic plants. (a) and (b) T‐DNA region of the plasmid pCAMBIA1300‐Ppht1::*ZmPIN1a* (±)‐P35S::*EPSP* and pCAMBIA1300‐Ppht1::*ZmPIN1b*(±)‐P35S::*EPSP*. The sense or antisense coding sequence of the *ZmPIN1a* or *ZmPIN1b* gene was inserted into the *Xbal*I site of the mini‐Ti plasmid. Ppht1, barley *Pht1* promoter (Schunmann *et al*., 2004); Tnos, a nos terminator; P35S and T35S, CaMV35S promoter and CaMV35S terminator; and *EPSP,* 5‐enolpyruvylshikimate‐3‐phosphate (EPSP) synthase; *ZmPIN1a* (+) or (−), *ZmPIN1b* (+) or (−) full cDNA in sense or antisense orientation. (c) PCR assay of T3 transgenic maize plants. Lane M, DNA marker DL2000; (+) was the PCR result of plasmid pCAMBIA1300‐Ppht1::*ZmPIN1a* (±)‐P35S::*EPSP* and pCAMBIA1300‐Ppht1::*ZmPIN1b* (±)‐P35S::*EPSP*; (−) was the PCR result of H_2_O; the lines used are those described in Figure 1. (d) and (e) Southern blotting analysis using a probe for *EPSP* (d) or *ZmPIN1a* (e) indicated that exogenous *ZmPIN1a* integrated in the maize genome. In all cases, genomic DNA (40 μg) of transgenic and WT plants was extracted and digested with *Bgl*II or *Eco* *R*V, which had one cut site in the T‐DNA region for Southern blotting hybridization. M presents the λDNA/*Eco*T14 molecular weight marker, (+) presents the positive control using mixture of plasmid DNA and the digested plasmid DNA (*Hind* III+S*ac* II, can release a 6.8‐kb fragment including T‐DNA and part of the plasmid, and the lines used are described in Figure 1. The blue asterisk indicates the exogenous band. (f) Dot blot hybridization with AtPIN1 antibody. (g) Western blot of plasma membrane protein hybridization with AtPIN1 antibody. The plasma membrane (PM), mitochondria (Mt, the precipitate after the 21 000 ***g*** centrifugation) and soluble protein of maize root segment (1 cm form root tip of the seminal roots) were segregated by centrifugation according to the method of Abas and Luschnig (Abas and Luschnig, 2010). (h) ZmPIN1a transient expression assays using onion epidermal cells to show subcellular localization on the plasma membrane.

Transgenic maize homozygous lines known as *ZmPIN1a* and *ZmPIN1b* sense or antisense lines were screened using PCR and real‐time RT–PCR. Lines with significantly changed gene expression levels were selected for use in subsequent molecular, phenotypic and physiological analyses (Figures 1 and 2). When cultured in vermiculite, there were no obvious differences between the *ZmPIN1b* transgenic and WT lines, whereas the *ZmPIN1a* sense lines were more dwarfed than the WT and antisense lines primarily because of the short lower nodes of the plants. After washing with tap water to remove the vermiculite, the *ZmPIN1a* sense lines had deeper roots, with longer seminal roots that had denser lateral roots. The root dry weights of the *ZmPIN1a* sense lines were higher than those of the WT and antisense lines (Figure 1f). Four of the six selected sense *ZmPIN1b* transgenic lines exhibited significantly enhanced biomass in roots, and the other two lines had values close to that of the WT line (Figure 1g). Although some differences from the WT line were found for all selected antisense lines, they were not statistically significant. However, *ZmPIN1b* has minor effects.

**Figure 3:**
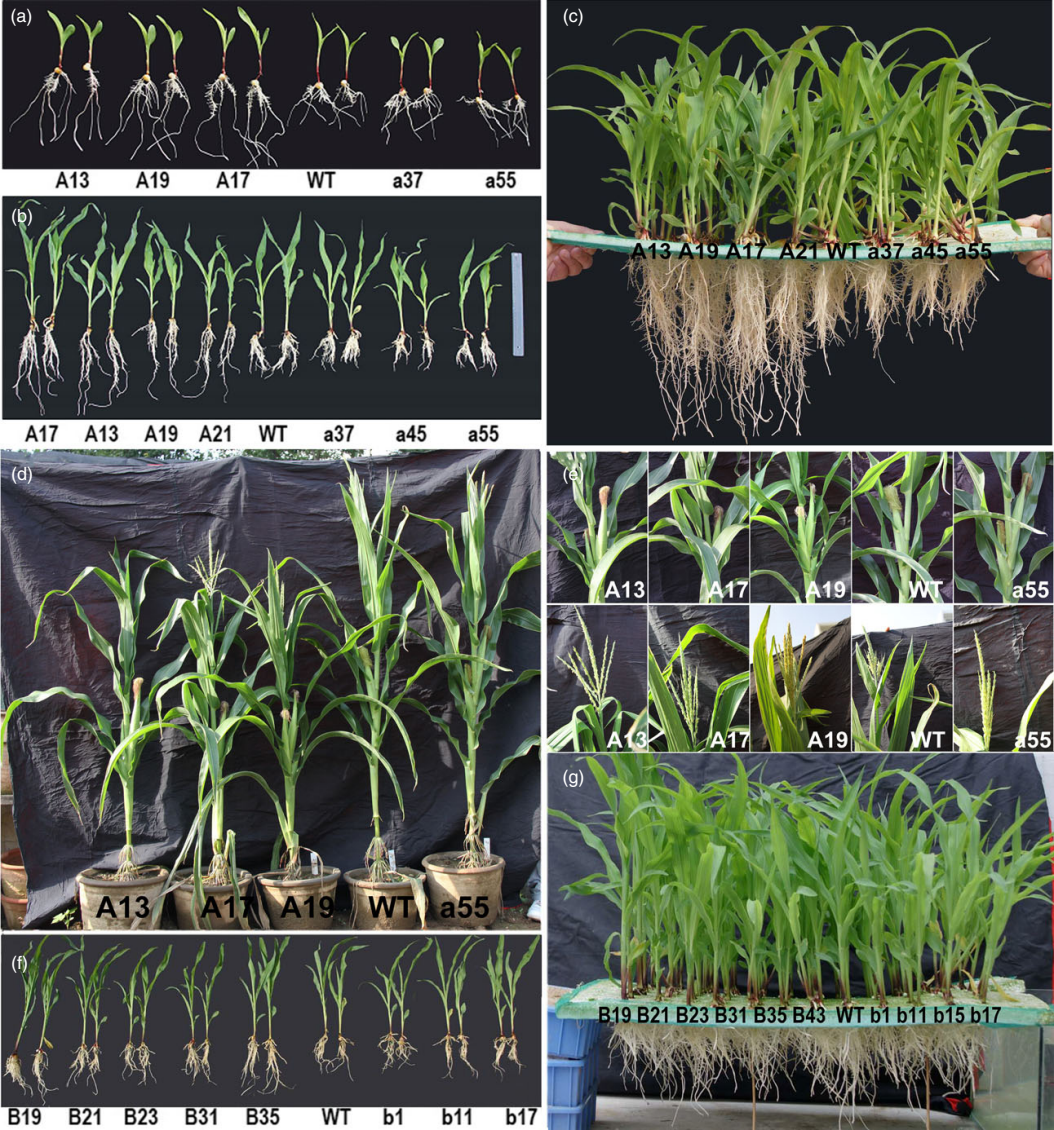
Phenotype of *ZmPIN1a* and *ZmPIN1b* transgenic lines. (a) and (b), (c) *ZmPIN1a* transgenic and WT lines cultured in nutrient solution for 3 and 12 days. (d) and (e) Plants, tassels and ears of *ZmPIN1a* transgenic and WT lines grown in large pots. (f) and (g) *ZmPIN1b* transgenic and WT lines cultured in nutrient solution for 12 days. The lines used are described in Figure 1.

The sense *ZmPIN1a* lines showed more developed root systems when cultured in nutrient solution, such as in vermiculite medium conditions. The root number of the sense lines was 129%–156% of that of WT plants, with no clear increase in the numbers of crown roots or seminal roots. The a45 antisense line had significantly fewer lateral roots, while the other antisense lines were close in value to the WT line (Figure 3 and Tables S2 and S3). Root–length determination showed that the lateral roots, seminal roots and whole roots of the sense *ZmPIN1a* lines were significantly longer than those of the WT and antisense lines (Figure 3 and Tables S2 and S3). An analysis of average root length demonstrated that *ZmPIN1a* overexpression increased the number of lateral roots, inhibited the elongation of lateral roots and resulted in longer seminal roots with short, dense lateral roots. To confirm the relationship of *ZmPIN1a* expression levels and the plant development, *ZmPIN1a* RNAi and UFMu mutant lines were obtained and observed (Figure S2). Constitutive knock down or knockout of *ZmPIN1a* can significantly inhibit root development in maize rather than just expressing the antisense *ZmPIN1a* driven by barley *Pht1,* a phosphate transporter 1 promoter (Figure 2) that is mainly expressed in roots (Schunmann *et al*., 2004). The sense Zm*PIN1b* lines exhibited a slightly more developed root system than the WT and antisense *ZmPIN1b* lines, and the dry weight of their roots was higher than that of WT line (Figures 1g, 3 and Table S2).

We monitored the growth of sense and antisense *ZmPIN1a* lines and WT plants that were cultured in large pots and in the field. At the vegetative stage, the sense *ZmPIN1a* lines exhibited no obvious differences from the WT line other than their lower plant height. At the reproductive stage, the plants of the sense lines were significantly shorter, at 73%–91% of the WT plant heights (Figure 3d and e). The heights of the 1st ear were 20–30 cm lower than the ear heights of the WT and antisense lines. There were 2–3 ears per plant in the sense lines. The lower ears did not have healthy development, and the WT and antisense lines had one ear each in the same culture conditions. No growth or maturation delays were found among these lines, and the anthesis‐silking interval of all sense and antisense lines was near the WT line values.

### Grain yields of transgenic lines under different plant densities

**Figure 4:**
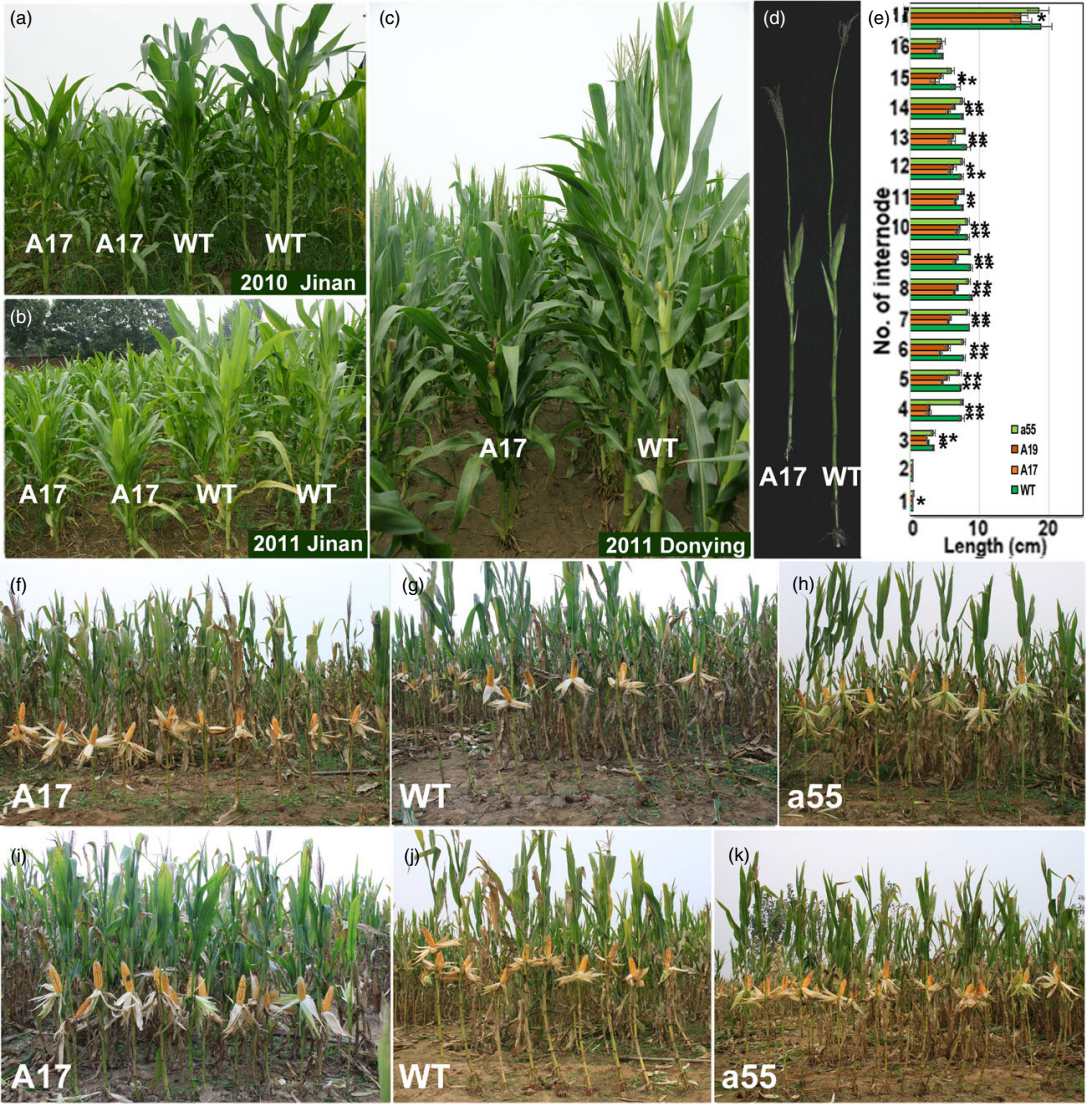
Yield performances of *ZmPIN1a* transgenic and WT lines under different density conditions. (a)‐(c) *ZmPIN1a* transgenic and WT lines were planted in different years and sites to demonstrate hereditary stability. (d) Stems of lines A17 (sense line) and WT. (e) Internode length of *ZmPIN1a* transgenic lines and WT plants. Values are the means ± SD. The asterisks indicate significant differences between the transgenic and WT lines at the *0.05 or **0.01 level using the *t*‐test (*n* = 5). (f)‐(h) Lines under moderate‐density culture. (i)‐(k) Lines under high‐density culture. The lines used are described in Figure 1.

Figure 4 shows the T4 generation plants at different densities in the field; the *ZmPIN1a* antisense and WT lines had longer internodes, and the *ZmPIN1a* sense lines exhibited short internodes and shorter plant heights. At a moderate density (73 370 plants/ha), the grain yield of the *ZmPIN1a* sense and WT lines displayed some differences, and the latter had a slightly higher grain yield per plant because there was one large ear per plant under moderate‐density cultivation. When planted at a high density (120 000 plants/ha), the grain yield per WT plant was reduced by an increased percentage of barren plants at this density. The *ZmPIN1a* sense lines maintained relatively better pollination and kernel development, and a higher yield per plant was obtained. Additionally, under this cultivation density, the rate of barren plants was much lower than that of the WT plants; therefore, the yield per plot was significantly higher than the yields for the WT and antisense lines (Table S4). Trials with different densities indicated that the sense *ZmPIN1a* lines were valuable in maize breeding for their deeper root systems, reduced plant height and ear height, higher yield per unit area, and especially in resisting lodging caused by wind and rain.

### *ZmPIN1a* overexpression improved resistance to drought stress and low‐phosphate starvation by creating an elongated root system

**Figure 5:**
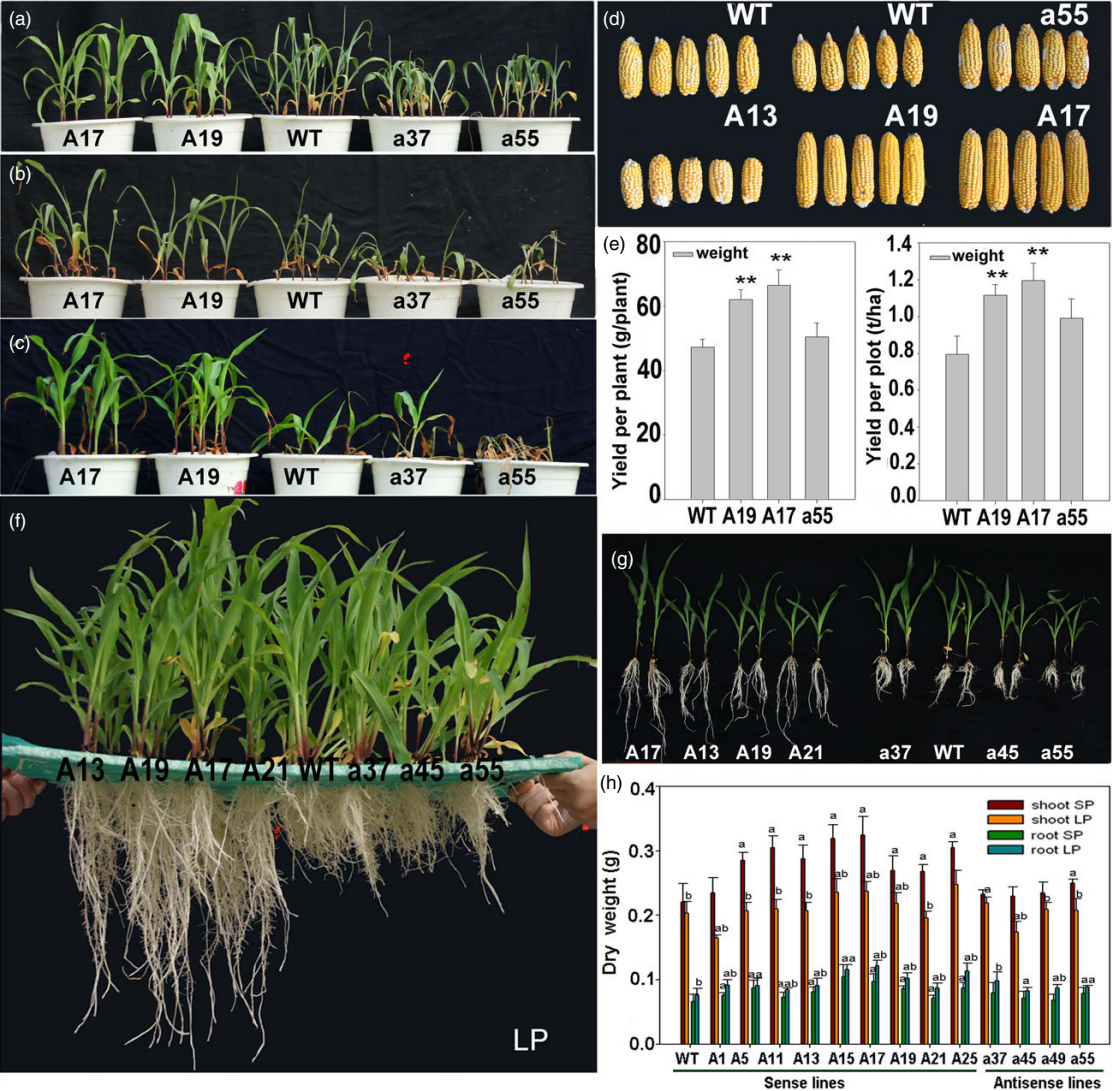
Improved tolerances for drought and low‐phosphate levels in *ZmPIN1a* transgenic lines. (a)–(c) *ZmPIN1a* transgenic lines and WT plants grown in soil pots for 2 days (a) or 5 days (b) without water and rewatered for 2 days (c). (d) and (e) Ears and yield of *ZmPIN1a* transgenic and WT lines suffering from drought stress in the field. Values are the means ± SD. The asterisks indicate significant differences between the transgenic and WT lines at the **0.01 level using the *t*‐test (*n *= 8). (f) and (g) *ZmPIN1a* transgenic and WT lines grown in LP solution. (h) Biomass analysis of *ZmPIN1a* transgenic and WT lines plants grown in SP and LP solutions. Values are means ± SD. An ‘a’ indicates a significant difference between transgenic and WT lines in the same nutrient solution, and ‘b’ indicates a significant difference in genotype under SP versus LP conditions at the 0.05 level using the *t*‐test (*n *= 10). The lines used are those described in Figure 1.

The drought resistance of the T4 and T5 generation transgenic plants was examined. As shown in Figure 5a–c, all lines showed leaf wilting caused by water shortage after 2 days without watering, but the sense lines were less wilted. All plants exhibited serious wilting after another 3 days without water. The WT and antisense lines showed severe dehydration and began to die, and sense lines A17 and A19 maintained better growth. After being rewatered, almost all the sense lines recovered to normal, but few WT or antisense plants survived. The plant yields suffered from drought stress in the field, demonstrating that sense lines A17 and A19 had higher yields per plant and per plot when compared with the WT line, especially A17, which yielded 147% of the WT value (Figure 5d–e). The robust root system of the plant and its shorter height aboveground ensured that it could absorb water and mineral nutrition from arid soil, which is important to sustain the normal activity of a living plant subjected to drought stress.

When cultured in LP solution, the WT plants under LP acclimation exhibited increased elongation in the seminal roots and reduced lateral roots, and the sense lines showed increased lateral roots and total root numbers, increased length of crown roots and seminal roots and reduced length of the lateral roots (Figure 5f and Tables S5 and S6). The number of lateral roots of the sense lines was 163%–192% that of the WT line, and the length was 99%–142% of the WT value. The increased total root length for the sense lines was primarily explained by their longer seminal roots (Figure 5f–h and Tables S5 and S6). The phenotype caused by *ZmPIN1a* overexpression was more significant under LP conditions, which may have been caused by the induction of low phosphate on root growth. The barley *Pht1*, the phosphate transporter *1* promoter, was used in the transgene, which was identified as responsive to phosphate deprivation (Schunmann *et al*., 2004).

### Morphological analysis of *ZmPIN1a* transgenic lines treated by different dosage of IAA, NAA and NPA

**Figure 6:**
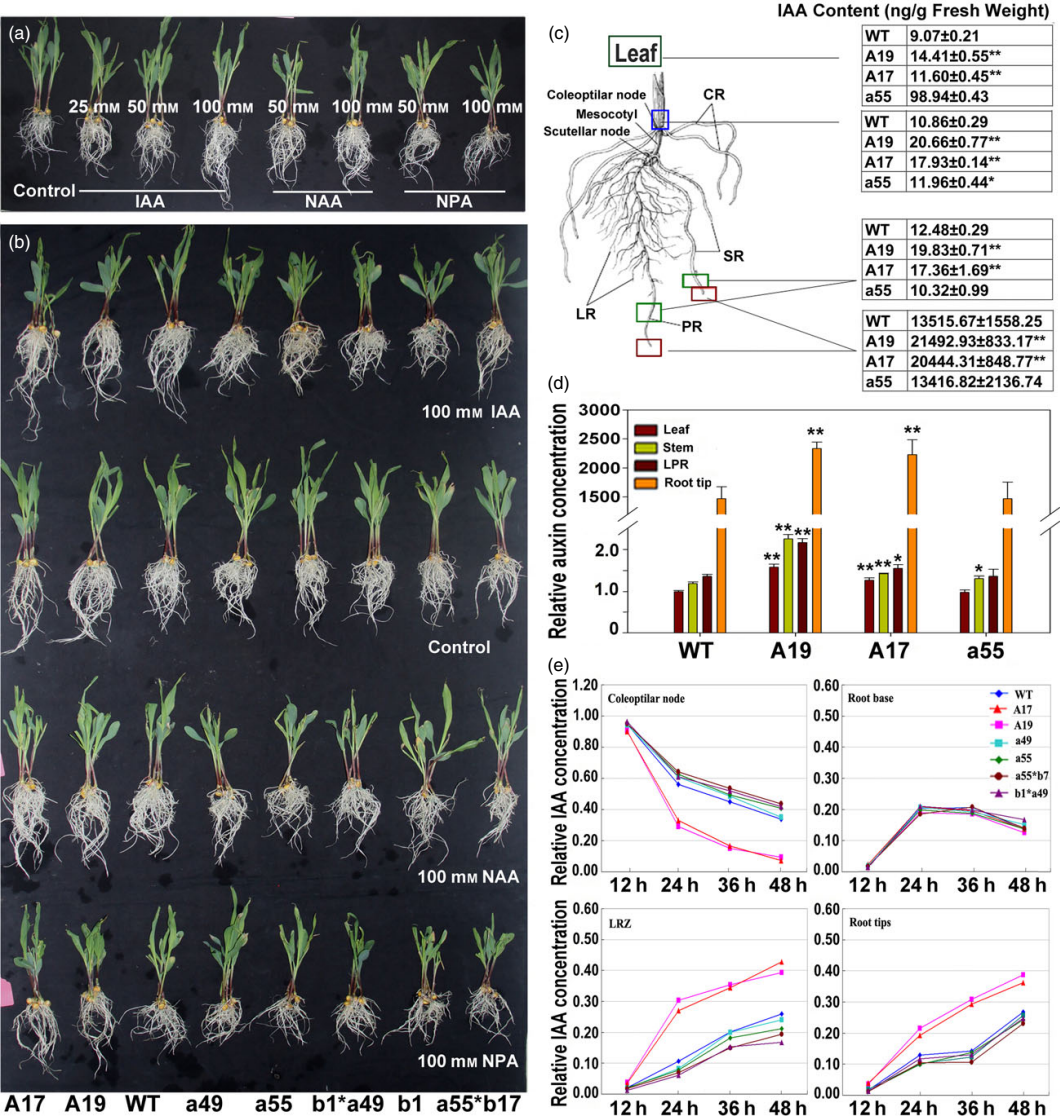
Variations in IAA concentrations and transport capacity in *ZmPIN1a* transgenic plants. (a) Morphological changes of WT plants after treatment with IAA, NAA, NPA and control treatment as presented in Figure S3 for 3 days. (b) The morphological changes of transgenic lines, transgene pyramid lines and WT plants after IAA, NAA, NPA and control treatment as presented in Figure S3 for 3 days. (c) Auxin concentration of the leaf, coleoptilar node, LRZ (the 1.0–1.5 cm segment with lateral root outgrowth) and root tips of *ZmPIN1a* transgenic and WT lines cultured in nutrient solution for eight days. The boxes showed the regions for auxin determination. (d) Relative auxin concentration in different parts of maize seedlings. The value of WT leaves was set as 1, and all data were compared to WT leaves. The values are means ± SD. The asterisks indicate significant differences between transgenic and WT lines at the *0.05 or **0.01 level using the *t*‐test (*n* = 5). (e) IAA transport capacity determination of *ZmPIN1a* transgenic and WT lines by distribution changes of ^3^H‐IAA. Relative IAA concentrations of coleoptilar nodes, root base, LRZ and root tips from *ZmPIN1a* transgenic, transgene pyramid lines and WT lines were calculated. Values are the average of three independent biological repeats as raw data and presented in Table S7.

To explore the root architecture modification mechanism of transgenic maize, an application of different dosages of IAA, NAA and NPA was performed at the shoot apex of seedlings (Figure 6a and b). Instead of socking the root into solution with auxin and monitoring the absorption and transport of auxin by the root system, we only dropped the auxin solution into the shoot apex to check the auxin transport capacity from shoot to root (Figure S3). IAA application promoted the elongation of primary and seminal roots with a dosage effect, and the growth of lateral root was inhibited. For NAA, which is independent of the polar auxin transporters of the plant, in addition to the developed growth of the primary, seminal root, the growth of lateral roots was also promoted. Auxin transport inhibitor (NPA) retarded the growth of roots (Figure 6a). When seedlings from the *ZmPIN1a* sense and antisense lines, the pyramiding lines of *ZmPIN1a* antisense, *ZmPIN1b* antisense and the WT line were exposed to IAA, NAA and NPA solution at the shoot apex (Figure 6b), the *ZmPIN1a* sense plants in these treatments showed a robust root system with a modified architecture as described above, and the application of IAA promoted the elongation of the primary and seminal roots of WT and antisense lines just as in the phenotype caused by *ZmPIN1a* overexpression (Figure 6a and b). The application of exogenous IAA and NAA had different biological effects on maize root development. IAA was restricted by a complex auxin transport system composed of different transporters, while the synthetic hormone NAA was verified to greatly increase cellulose fibre formation in plants without selection of cell types. Here, an increase in IAA transport to roots through overexpression of auxin transporter (*ZmPIN1a*) or alteration of the homeostasis of the auxin sink and pool (application of IAA to WT plants) leads to a developed root system with longer seminal roots and denser lateral roots.

### Overexpression of *ZmPIN1a* enhanced IAA transport from shoot to root

As shown in Figure 1, introduction of *ZmPIN1a,* which was driven by the barley *Pht1,* the *1* phosphate transporter promoter, led to increased *ZmPIN1a* expression in the maize plant, especially the root. The polar auxin transport genes seemed to establish a new homeostasis of auxin transport. As ZmPIN1a can be successfully targeted to the plant plasma membrane (Figure 2f–h), we wondered whether this led to the alteration of auxin distribution and auxin concentration. Local auxin concentrations of maize plants were determined at the two‐leaf stage from different lines (Figure 6c). The concentrations of auxin exhibited impressive increases in the root tips. As shown in Figure 6c and d, the auxin concentrations of leaves, coleoptilar node and LRZ were approximately one‐third higher in the sense lines than the WT and antisense lines, and in the root tips, there were 1.59‐ and 1.52‐fold higher concentrations in the A19 and A17 sense lines compared with the WT line and antisense line a55, respectively. We concluded that the increased local auxin concentration of the root tips in *ZmPIN1a* sense plants (Figure 6c and d) promoted the elongation of primary roots and inhibited the growth of lateral roots.

To understand why the auxin concentration in the root tips of *ZmPIN1a* sense plants substantially increased, we determined the IAA transport capacity of *ZmPIN1a* sense lines, the antisense line and the WT line using ^3^H‐IAA. The sense lines transported more ^3^H‐IAA to the root system, especially in the meristematic zone 48 h after treatment, that is, the increased expression of *ZmPIN1a‐*enhanced auxin transport from the apical meristem of the shoot to the root led to the accumulation of auxin in the root tips (Figure 6e and Table S7). For the pyramiding lines of *ZmPIN1a* antisense and *ZmPIN1b* antisense lines, weaker transport capabilities and more underdeveloped root systems were observed. This suggested that auxin transporters are functionally redundant and that overexpression of *ZmPIN1a* increased the IAA transport capacity from shoot to root, leading to a local auxin concentration increase in the root. The alteration of the local auxin concentration boosted root growth and phenotypic modification.

### Changes in the transcriptome by altering *ZmPIN1a* expression

**Figure 7:**
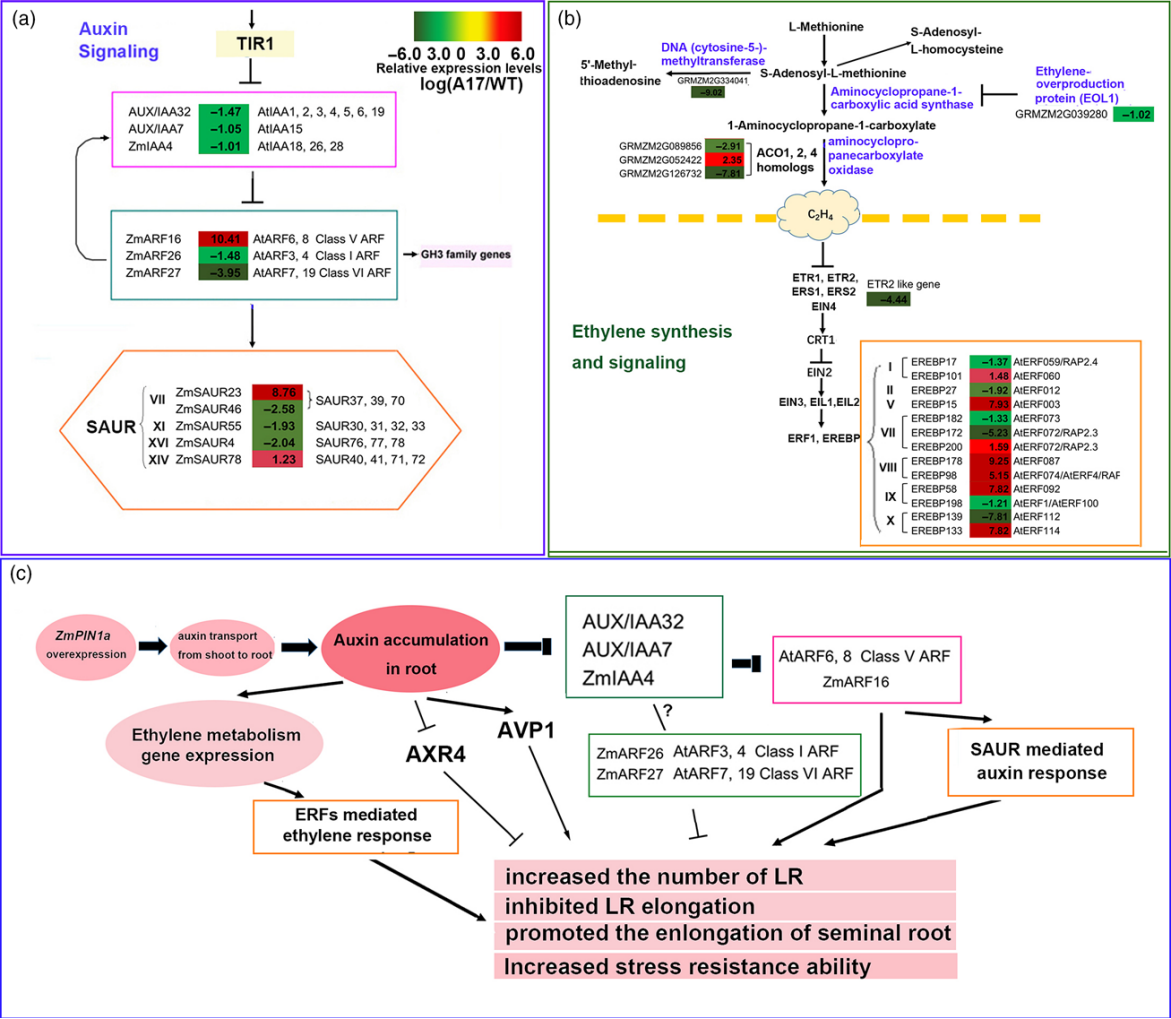
*ZmPIN1a* regulated auxin, ethylene biosynthesis and signalling and model summarizing effect of ZmPIN1a on root morphology in maize. (a) Genes involved in auxin signalling differentially expressed between the *ZmPIN1a* overexpression A17 and WT lines. (b) Genes involved in ethylene biosynthesis and signalling differentially expressed between the *ZmPIN1a* overexpression A17 and WT lines. The numerical values are the log2 ratios (A17/WT) of the DGEs, and the background colour showed the gene relative expression levels compared with the WT line (red represents up‐regulated, green is down‐regulated, and yellow is no significant change). Bold type means the difference meets the criterion for the log2 ratio (A17/WT) <‐1 or ratio >1 and *P* < 0.001. (c) Model summarizing the main results regarding *ZmPIN1a* overexpression on root morphology changes in maize.

A genome‐wide transcriptomic analysis (GEO: GSE57291) showed that altering *ZmPIN1a* expression led to wide‐ranging gene expression changes in roots, especially for auxin and ethylene signalling. When comparing the sense line A17 with the WT line, Aux/IAA‐ARF‐dependent regulation changed. As shown in Figure 7 and Table S8, the *AUX/IAA32* (orthologue of *AtIAA1/2/3/4/5/6/19*), *AUX/IAA7* (orthologue of *AtIAA15*) and *ZmIAA4* (orthologue of *AtIAA18/26/28*) were down‐regulated by 0.36‐, 0.48‐ and 0.50‐fold, respectively, in the root of sense line A17 compared with the WT line. *ZmARF16* (orthologue of *AtARF6/8*, Class V ARFs) was 1359‐fold up‐regulated, while another two ARFs, *ZmARF26* (orthologue of *AtARF3/4*, Class I ARFs) and *ZmARF27* (orthologue of *AtARF7/19*, Class VI ARFs), were down‐regulated by 0.36‐ and 0.06‐fold, respectively. ARF6/8 was reported as a positive regulator of root development, *AtARF3/4* was a negative regulator of root development, and *AtARF7* and *19* redundantly regulated lateral root formation (Guilfoyle and Hagen, 2007; Lavenus *et al*., 2013; Peret *et al*., 2009). The dramatic induction of *ZmARF16* (orthologue of *AtARF6/8*) and the reduction of *ZmARF26* (orthologue of *AtARF3/4*) and *ZmARF27* (orthologue of *AtARF7/19*) may largely contribute to the morphology changes in *ZmPIN1a* sense lines. In addition to AUX/IAA‐ARFs changes, other rapidly and transiently auxin‐induced small auxin‐up RNA (SAUR) family genes significantly changed. Two SAUR genes belonging to the SAUR37/39/70 Class VII subgroup, which had been reported in calcium signalling, were changed, with a 435‐fold increase in GRMZM2G365188 and a 0.17‐fold reduction in AC196708.3_FGT006. *ZmSAUR55* and *ZmSAUR4* were down‐regulated by 0.26‐ and 0.24‐fold, respectively, and their orthologues in Arabidopsis have been reported as negative regulators of plant growth. *ZmSAUR78* was 2.35‐fold up‐regulated, and overexpression of *SAUR41* (orthologue in Arabidopsis) promotes hypocotyl elongation as a result of increased cell expansion (Kong *et al*., 2013; Li *et al*., 2015; Ren and Gray, 2015; Spartz *et al*., 2012). It seems that the *ZmPIN1a* overexpression led to a change in the local auxin gradient concentration, and this change altered auxin‐ signalling transduction; finally, auxin‐ mediated root development and growth. Other key genes involved in root development were remarkably changed in the sense line, including *H* ^*+*^ *‐translocating inorganic pyrophosphatase* (*AVP1*, GRMZM2G041065) (643.6‐fold up‐regulated) and the auxin response mutant (*AXR4*) coding gene (0.002‐fold down‐regulated). *AVP1* overexpression increased cell division and promoted root growth (Schilling *et al*., 2017), while the mutation of *AXR4* was defective in root gravitropism and reduced lateral root number (Hobbie and Estelle, 1995).

In addition to auxin signalling, ethylene synthesis and signalling were altered. As shown in Figure 7 and Table S8, *S‐adenosylmethionine decarboxylase* (GRMZM2G060369) was 265‐fold up‐regulated, and three aminocyclopropanecarboxylate oxidase genes (ACO) were differentially expressed, with 0.13‐fold down‐regulated GRMZM2G089856, 0.004‐fold down‐regulated GRMZM2G126732 (ACCO20) and 5.10‐fold up‐regulated GRMZM2G052422 (ACCO35). The ethylene receptor ETR2‐like gene (GRMZM2G075368) was significantly reduced in roots (0.0025‐fold) in the sense lines compared with the WT line. In addition, seven EREBP factors were dramatically induced and six other EREBP factors were dramatically down‐regulated, including the homologues of RAP2.4, RAP2.3, AtRFF1 and AtERF4. The altered local auxin gradient concentration led to the alteration of the metabolism and the signalling process of other plant hormones, especially ethylene. All of this contributes to the growth and stress response changes caused by *ZmPIN1a* overexpression.

## Discussion

### Increased IAA concentration in maize roots promotes root growth and dwarfs plant height

Auxin has been shown to play a central role in many aspects of plant morphogenesis and response to environmental stimulations. Over the past decade, several auxin‐related mutants have been identified, and some mechanisms of auxin action have been revealed, particularly auxin polar transport in *Arabidopsis*. In Arabidopsis root, auxin, as an integrator of endogenous and exogenous signals for root development, controls lateral root development through multiple auxin‐signalling modules. Indeed, many hormones or nutrients known to influence LR development have been found to do so by interfering with auxin homeostasis (synthesis, conjugation and degradation), transport or response (Lavenus *et al*., 2013). Evidence from many studies highlights the central role of auxins in orchestrating the final root architecture from auxin synthesis to signalling. Lateral root meristem activation and elongation are dependent on auxin. Exogenous application of auxin induces cell division and results in lateral root initiation in the pericycle at the xylem poles in Arabidopsis. IAA induces adventitious roots AR initiation through the activation of a coordinated efflux/influx involving PIN1 and AUXIN1 (AUX1), which causes the IAA gradient essential for induction in Arabidopsis. Moreover, the influx carrier LIKE‐AUX1‐3 (LAX3) is also essential for AR emergence (Della Rovere *et al*., 2013). In this study, overexpression of ZmPIN1a elevated IAA concentrations in roots and increased the number of lateral roots, as well as the length of seminal roots that bore dense short lateral roots. It also improved root weight, while both plant and ear heights were significantly shortened. Application of external IAA and the determination of IAA transport capacity indicated that the increased expression of *ZmPIN1a* enhanced auxin transport from the shoot apex to the root and led to auxin accumulation in the root tips to modify the root architecture. It can be inferred that elevated IAA concentrations in roots facilitated changes in the expression levels of many genes involved in phytohormone metabolism and the signalling cascade, producing a modified root system and shorter plants.

### Root morphology alteration of the sense line resulted from expression changes of genes in auxin signalling and root development

A genomewide transcriptome analysis showed that altering *ZmPIN1a* expression led to wide‐ranging gene expression changes (GEO: GSE57291). Comparative expression analysis of genes revealed IAA28‐ARFs, which mediated cell specification, ARF6/8, which regulated cell elongation, and ARF3/4, which controlled lateral root growth (Guilfoyle and Hagen, 2007; Lavenus *et al*., 2013; Overvoorde *et al*., 2010), all of which showed significant changes in the sense line. The down‐regulated genes of *AUX/IAA32* (orthologue of *AtIAA1/2/3/4/5/6/19*), *AUX/IAA7* (orthologue of *AtIAA15*), *ZmIAA4* (orthologue of *AtIAA18/26/28*), *ZmARF26* (orthologue of *AtARF3/4*) and *ZmARF27* (orthologue of *AtARF7/19*) and the dramatically up‐regulated gene of *ZmARF16* (orthologue of *AtARF6/8*), all implied that auxin signalling is pivotal in modifying root growth and dwarfing plant height. However, their interaction, regulation of root growth, lateral root initiation and elongation must still be elucidated.

The dramatically induced expression of *AVP1* (positive regulator of root growth (Schilling *et al*., 2017)) and reduced expression of *AXR4* (negative regulator of root development (Hobbie and Estelle, 1995)) may contribute to the root morphology alteration under nonstress and various abiotic stress conditions, as in *Arabidopsis*. Moreover, high auxin levels led to the alteration of ethylene synthesis and signalling pathways through increased transcription of *S‐adenosylmethionine decarboxylase* (GRMZM2G060369), the regulation of *ACC* expression, the inhibition of the expression of the ethylene receptor *ETR2*‐like gene and the change in expression of dozens of *AP2*/*EREBP* transcription factors for ethylene. Auxin and ethylene act synergistically to reduce primary root elongation, but antagonistically in lateral root formation (Alarcon *et al*., 2014) and may be conserved in maize.

As summarized in Figure 7, the root morphology change from *ZmPIN1a* overexpression in roots was due to increased auxin local concentrations in the root. The activity of auxin signalling (IAA/ARF, SAUR) and key auxin‐regulated genes in root development (AVP1 and AXR4) and alteration of the ethylene synthesis signalling pathway also contribute to the increased stress response.

### *PIN* genes in maize show differential function

Notably, the homologous genes caused distinct biological effects on plant morphology and development. Most single Arabidopsis genes have several homologues in the monocotyledon crops rice and maize. Functionally redundant genes have different expression profiles or respond to different stimuli, which may be the result of increased gene members from one ancestor gene by divergent evolution. The Arabidopsis *pin1* mutant does not initiate any flowers, resulting in a naked inflorescence stem (Benkova *et al*., 2003; Galweiler *et al*., 1998; Reinhardt *et al*., 2003) Induced XVE‐*PIN1* Arabidopsis plants show twisted growth along the vertical axis in the hypocotyls of dark‐grown seedlings and are usually more pronounced close to the hypocotyl base. DR5::GUS staining of the XVE‐*PIN1* line after oestradiol treatment suggested stronger auxin accumulation in the root tip, which explains the root gravitropism phenotype (Mravec *et al*., 2008; Petrasek *et al*., 2006). Constitutive alteration of *OsPIN1* expression (a homologous gene of maize *ZmPIN1b* and *ZmPIN1c*) changes the tiller number and shoot/root ratio, especially for adventitious root emergence and development (Xu *et al*., 2005). In our study, both transgenes *ZmPIN1a* and *ZmPIN1b* were driven by the barley *Pht1* promoter (Schunmann *et al*., 2004). They showed different contributions to plant morphology; root systems with increased root depth were observed in *ZmPIN1a* sense lines, and increased adventitious roots were observed in the *ZmPIN1b* sense lines. Why are there different roles in the root development of maize? A sequence alignment showed that the primary difference among AtPIN1, OsPIN1a, OsPIN1c, ZmPIN1a and ZmPIN1b is the amino acids located in the large central hydrophilic loop. PIN polar localization is regulated by reversible phosphorylation of the hydrophilic loop (Huang *et al*., 2010; Michniewicz *et al*., 2007). Phosphorylation site prediction showed that two candidate phosphorylation sites were absent in ZmPIN1a (Figure S4). This finding suggests that different amino acids in the hydrophilic loop may influence the modification of proteins.

Promoter analysis indicated that *ZmPIN1a* and *ZmPIN1b* genes have different roles in plant development and environmental stimulus responses (Table S9). Examination of their expression patterns showed that *ZmPIN1b* was highly expressed in leaves (similar to OsPIN1a), whereas *ZmPIN1a* was primarily expressed in roots at levels lower than that of *ZmPIN1b* (Figure 1). These phenotypes indicate that PIN1 action in monocotyledons is more sophisticated and must still be elucidated, especially the relationships between gene expression patterns, protein modifications, local auxin concentrations, biological effects and alterations in plant morphology.

### An ideal maize phenotype for highly dense cultivation could be produced by *ZmPIN1a* overexpression

For maize breeding, a deep and vigorous root system enhances the plant's ability to absorb water and mineral elements and makes plants resistant to lodging caused by wind and rain. Moreover, *ZmPIN1a* overexpression enhanced resistance to drought stress and low‐phosphate starvation, thereby sustaining a higher yield compared to the WT line by promoting root elongation. The improved root system for *ZmPIN1a* sense lines enables the absorption of water and nutrients, and/or increased resistance to lodging (mainly contributed by deeper roots) and drought and low‐phosphate stress, which were very important for the yield improvement of maize under high‐density cultivation. Moreover, the auxin‐mediated response to abiotic/biotic stress was activated by overexpression of *ZmPIN1a*. This approach is a successful case of root genetic engineering and offers a new strategy and germplasm for ideal maize phenotype selection.

Agronomic interest in short plants derives largely from their ability to resist lodging caused by wind, rain or higher density, which allows them to effectively convert increased fertilizer input into higher yields. This notion is best exemplified by the success of the green revolution, which was made possible by the advent of dwarf wheat and rice varieties (Salamini, 2003). Reduced plant height is usually achieved by blocking the biosynthesis and signalling of gibberellins. The A13 lines, which have higher transgene expression levels, have reduced their plant height by a large margin via transport of more auxin to the root meristem from the shoot apex. Although this line has a similarly robust root system that increased resistance to drought and low‐phosphate stress, the restraint of shoot growth and multi‐ear production is adverse to maize yields. A robust root system and increased resistance to drought and low‐phosphate stress were observed in A17 and A19, and the appropriate reduction in plant height was associated with an increased yield per plant and per plot under higher density planting; lodging resistance was also enhanced. There is competition and restriction in plant roots versus shoot growth and in yield versus resistance to environmental stress. It appears that refined regulation maintains growth, reproduction and resistance to various stresses in plants.

## Experimental protocol

### Plant materials

The plant materials used in this study were the maize elite inbred line DH4866 and its transgenic homozygous lines derived from independent T_0_ plants (Figures 1 and 2). T3 generation seedlings were used for morphological analysis in vermiculite, soil in pots and hydroponic cultures. T4 and T5 generation plants were used for drought stress and yield analysis in the field. For morphological analysis of the *ZmPIN1a* RNAi lines, T2 generation seedlings were used. For the UFMu mutant analysis, the *ZmPIN1a* UFMu mutants were kindly provided by the maize stock centre, and the homozygous plants were generated by self‐pollination.

### Measuring plant morphology and biomass (details in Data S1)

Hydroponic cultures, LP treatment and root morphology analysis were performed as described by Li *et al*. (2011). The roots and shoots were dried in an oven at 80 °C to a constant weight and then weighed. Drought treatment plants in soil pots at the three‐leaf stage grown under conditions of 32/25 °C (day/night) at a photon flux density of 700 μmol/m^2^/s (14‐h light/10‐h dark) were denied water, and rain was avoided. The plant parameters were recorded at days 2 and 5 of the drought treatment and 2 days after recovery.

### Gene clones, sequence analysis and expression analysis

The cDNA of the maize elite inbred line DH4866 was used for gene clones (details in Data S1). Gene‐specific primers were designed as the submitted sequences (Table S1). Sequence analysis was performed with Clustal W2 and MEGA 5 (Tamura *et al*., 2011). Phosphorylation site prediction was performed with KinasePhos (Huang *et al*., 2005). Promoter searches for cis elements were performed using the PlantCARE website (Lescot *et al*., 2002).

Maize total RNA was extracted from the samples using TRIzol reagent, treated with RNase‐free DNase and used for cDNA synthesis. Real‐time RT–PCR was performed in an ABI7300 with a SYBR Green RT–PCR Kit (Takara, China) according to the manufacturer's protocol. The gene transcript levels were calculated using the 2^−ΔΔCt^ method (Livak and Schmittgen, 2001) except with a special footnote, and maize *Actin1* (NM_001155179.1) was used as an internal control. The entire experiment was repeated three times, and the primers used in this study are shown in Table S1.

### Producing and identifying the transgenic lines (details in Data S1)

The sense or antisense coding sequences of *ZmPIN1a* or *ZmPIN1b* were inserted in the mini‐Ti plasmid pCAMBIA1300‐Ppht1:: MCS‐ P35S::*EPSP* for maize genetic transformation. The transgene was driven by the barley *Pht1* promoter (Schunmann *et al*., 2004) and the *5‐enolpyruvylshikimate‐3‐phosphate* (*EPSP*) *synthase* gene as a selectable marker gene driven by the CaMV35S promoter (Figure 2). For the *ZmPIN1a* RNAi lines, PCR fragments of *ZmPIN1a* were used to generate the hairpin structures. Both the gene fragment and selected marker gene *bar* were driven by CaMV35S promoter (P35S). The pCAMBIA1302‐Ppht1::*ZmPIN1a*‐*GFP* plasmid was used for transient expression assays in onion epidermal cells.

*Agrobacterium*‐mediated maize shoot‐tip genetic transformation was performed as described by a Chinese patent (CN 1305005A) (Zhang *et al*., 2001). Genomic DNA extraction, PCR assay and Southern blotting (genome DNA digested with *Bgl*II for EPSP probe and *EcoR*V for *ZmPIN1a* probe) were performed as described by Li *et al*. (2011). For Western blotting, an anti‐AtPIN1 antibody (aP20 sc‐27163, Santa Cruz Biotechnology) was used. Plasma membranes were isolated according to the method of Abas and Luschnig (2010). The mitochondrial fraction was the precipitate of the supernatant using 21 000 ***g*** centrifugation. Before loading, protein solutions were determined with Coomassie brilliant blue stain method, and equal amounts of protein were loaded on gel for each sample.

### Determining the IAA concentration and IAA transport capacity determination

Maize plants in hydroponic cultures at the two‐leaf stage were used for IAA concentration determination. Samples were prepared according to the method of Edlund *et al*. (1995). The analysis was performed using a gas chromatograph–mass spectrometer (GC‐TOF MS; Agilent/LECO) fitted with a capillary column (DA‐35 ms; 30 m × 0.25 mm × 0.25 μm; Agilent).

For IAA transport capacity determination (details in Data S1), maize plants at the two‐leaf stage were used. After being cut and nicked, the hormone solution (^3^H‐IAA, IAA, NAA or NPA) was slowly dripped onto the shoot apex. After infiltration, the plants were cultured for IAA transportation or morphology analysis. The ^3^H‐IAA contents in different parts of the plant were calculated based on fresh weight.

### Yield determination of drought and density gradient experiments in the field (details in Data S1)

Field experiments for the drought and density gradient were performed in the experimental fields of Jinan (drought and density gradient, 117°29′E, 36°54′N) and Dongying (density gradient, 118°49′E, 37°46′N) in the maize growing season. The trial plots were arranged in a randomized complete block design with four replications (five rows per plot for density gradient and two rows per plot for drought experiment). Plants were thinned at the three‐leaf stage to ensure the scheduled densities (moderate density, 73 370 plants/ha, high density, 120 000 plants/ha and 66 700 plants/ha for drought). For the drought treatment, at the 10‐leaf stage, the plants were subjected to drought stress for six weeks, with soil water content maintained from 15% to 17% at a depth of 40 cm during treatment. Mature ears were harvested to determine kernel yield, ear length and number of kernel rows. The experiment was repeated over 2 years.

### DGE analysis of *ZmPIN1a* transgenic lines and wild‐type control

Total RNA was extracted from the leaves and roots of *ZmPIN1a* transgenic and WT lines at the two‐leaf stage. Tag preparation, DNA purification and Illumina sequencing were performed by BGI Tech Solutions Co., Ltd. (Shenzhen, China) according to the standard procedure. Bioinformatics analysis for digital gene expression profiling was performed according to the bioinformatics analysis procedure of BGI Tech.

### Data analysis and biostatistics

All values are depicted as the average ± SD (standard deviation) from at least three independent experiments. Sample sizes are provided in the figure legends and table legends. For biomass, root morphology, IAA content and yield analysis, significant differences between transgenic and WT lines were assessed using Student’ s *t*‐test (*t*‐test, two‐sided).

## Author Contributions

Design: Zhang, J.R. & Li, Z.X.; execution of experiments: Li, Z.X., Zhang, X.R., Li, Y.J., Peng, Z.H., Zhao, Y.J., & Zhang, G.F.; analyses: Li, Z.X. & Zhang, J.R.; interpretation of results: Li, Z.X., Zhang, J.R., Zhang, X.R.; writing: Li, Z.X.; critical reading: Zhang, J.R.; project management: Zhang, J.R.

## Competing Financial Interests

The authors declare no competing financial interests.

## Supporting information


**Figure S1** Phylogenetic tree, synteny analysis of PIN1 in maize, rice and Arabidopsis.
**Figure S2** Molecular identification and morphology analysis of *ZmPIN1a* RNAi and UFMu mutants.
**Figure S3** The protocol of IAA transport capacity determination by using ^3^H‐IAA and IAA, NAA, NPA treatment.
**Figure S4** The sequence alignment and phosphorylation site prediction of ZmPIN1a, ZmPIN1b, OsPIN1a, OsPIN1c and AtPIN1.
**Table S1** PCR primers for *ZmPINs*’ expression analysis and the identification of transgenic plants.
**Table S2** Biomass of *ZmPIN1a* and *ZmPIN1 b* transgenic lines and wild type control in nutrient solution
**Table S3** Root morphology analysis of *ZmPIN1a*/*b* lines and the wild type control.
**Table S4** Yields under moderate and high density planting in the fields.
**Table S5** Root number of *ZmPIN1a* lines and WT plants cultured in nutrient solutions with SP or LP.
**Table S6** Root length of *ZmPIN1a* lines and WT cultured in nutrient solutions.
**Table S7** Determination of ^3^H‐IAA radioactive in *ZmPIN1a* transgenic lines and WT controls.
**Table S8** Differentially expressed genes involved in plant hormone metabolism and the signaling process in the root.
**Table S9** Promoter analysis of the *ZmPIN1a* and *ZmPIN1b* gene.

**Data S1** Supplementary Experimental protocol.
