# Supplementary material for: Enhancing auxin accumulation in maize root tips improves root growth and dwarfs plant height
Source: Plant Biotechnol J. 2017 Jun 23;16(1):86–99. doi: 10.1111/pbi.12751 (PMC5785362; doi:10.1111/pbi.12751)
Supplement: Supplementary file 1 — Figure S1 Phylogenetic tree, synteny analysis of PIN1 in maize, rice and Arabidopsis. Figure S2 Molecular identification and morphology analysis of ZmPIN1a RNAi and UFMu mutants. Figure S3 The protocol of IAA transport capacity determination by using 3H‐IAA and IAA, NAA, NPA treatment. Figure S4 The sequence alignment and phosphorylation site prediction of ZmPIN1a, ZmPIN1b, OsPIN1a, OsPIN1c and AtPIN1. Table S1 PCR primers for ZmPINs’ expression analysis and the identification of transgenic plants. Table S2 Biomass of ZmPIN1a and ZmPIN1 b transgenic lines and wild type control in nutrient solution Table S3 Root morphology analysis of ZmPIN1a/b lines and the wild type control. Table S4 Yields under moderate and high density planting in the fields. Table S5 Root number of ZmPIN1a lines and WT plants cultured in nutrient solutions with SP or LP. Table S6 Root length of ZmPIN1a lines and WT cultured in nutrient solutions. Table S7 Determination of 3H‐IAA radioactive in ZmPIN1a transgenic lines and WT controls. Table S8 Differentially expressed genes involved in plant hormone metabolism and the signaling process in the root. Table S9 Promoter analysis of the ZmPIN1a and ZmPIN1b gene. [file PBI-16-86-s002.pdf]

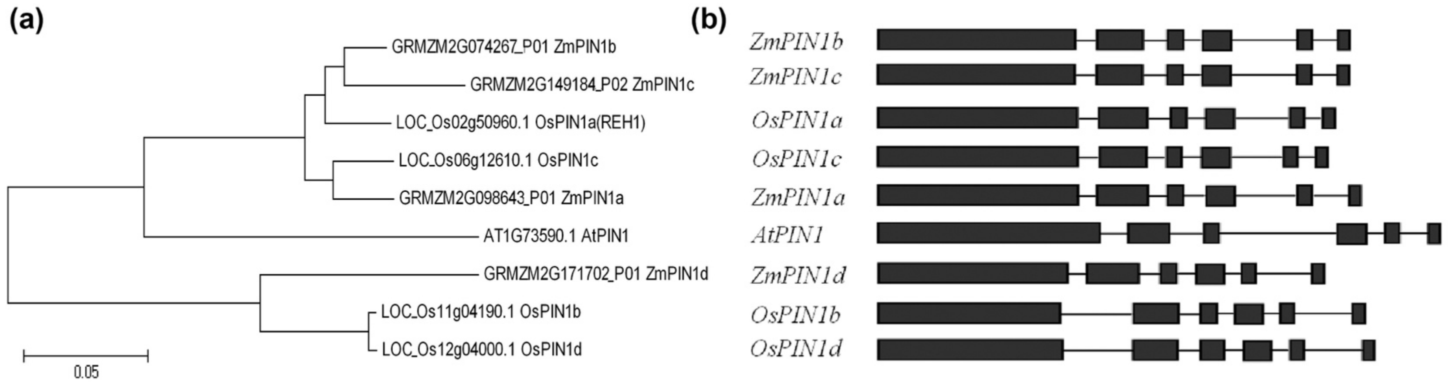

(c)

Name_Gene	Name_UniProtK	Transcript ID	Length (bp)	Protein ID	Length (aa)	Chromosome location	Name_Gene	homologs in rice	Chromosome location
ZmPIN1a	Q0MWY2_MAIZE	GRMZM2G098643_T01	2566	GRMZM2G098643_P01	601	Chr 9: 3,650,766-3,654,174 forward strand.	OsPIN1c	LOC_Os06g12610 (LOC_Os06g12610)	6:6865394-6868522
ZmPIN1b	Q0MWY1_MAIZE	GRMZM2G074267_T01	2684	GRMZM2G074267_P01	595	Chr 5: 206,727,149-206,730,565 forward	OsPIN1a	REH1 (LOC_Os02g50960)	2:31153034-31156193
ZmPIN1c	B2D1S1_MAIZE	GRMZM2G149184_T02	2299	GRMZM2G149184_P02	597	Chr 4: 182,007,439-182,010,555 forward	OsPIN1a	REH1 (LOC_Os02g50960)	2:31153034-31156193
ZmPIN1d	cl464_-1	GRMZM2G171702_T01	2093	GRMZM2G171702_P01	580	Chr 4: 186,642,791-186,645,569 reverse		No homologues	
Novel	Novel	GRMZM2G403601_T01	812	GRMZM2G403601_P01	267	Chr 3: 20,112,760-20,113,665 forward strand.	OsPIN1b	LOC_Os11g04190 (LOC_Os11g04190)	11:1701179-1704478
Novel	Novel	GRMZM2G403601_T01	812	GRMZM2G403601_P01	267	Chr 3: 20,112,760-20,113,665 forward strand.		LOC_Os12g04000 (LOC_Os12g04000)	12:1657206-1659737

Supplementary figure 1 Phylogenetic tree, synteny analysis of PIN1 in maize, rice and Arabidopsis

(a) Phylogenetic tree of PIN1 genes in Arabidopsis, rice and maize. Sequence analysis showed that four PIN1 orthologous genes are present in rice and maize, named ZmPIN1a (601 amino acids), ZmPIN1b (595 amino acids), ZmPIN1c (597 amino acids) and ZmPIN1d (580 amino acids). The main difference among the four is the amino acids located in the large central hydrophilic loop (from amino acid 290~414 of ZmPIN1a). (b) Intron and extron arrangements of PIN1 genes in Arabidopsis, rice and maize. (c) Length, location and synteny analysis of PIN1 genes in maize and rice.

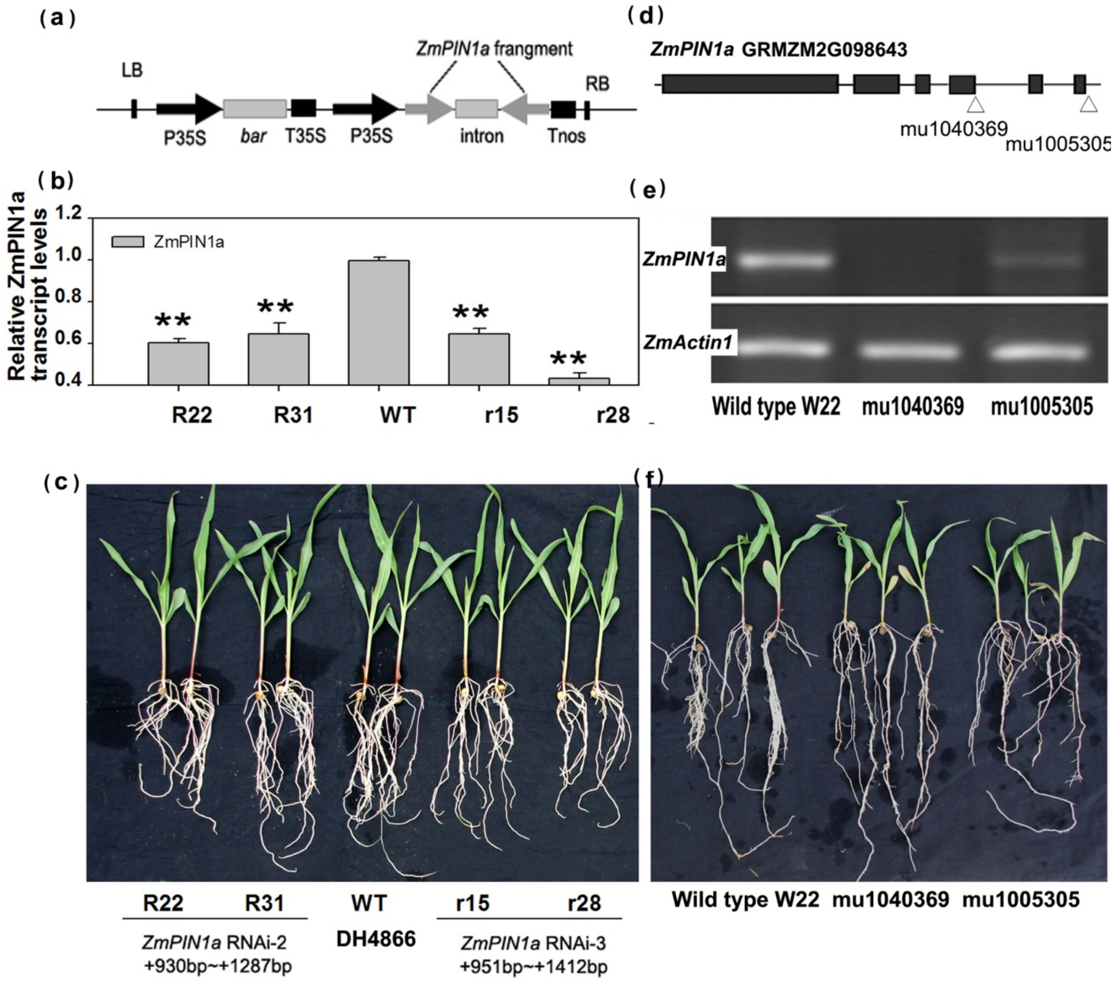

Supplementary figure 2 Molecular identification and morphology analysis of *ZmPIN1a* RNAi and UFMu mutants

(a) T-DNA region of the *ZmPIN1a* RNAi structure. In *ZmPIN1a* RNAi-2 the PCR fragment of +930bp~+1287bp was used, and in *ZmPIN1a* RNAi-3 the fragment of +951bp~+1412bp was used to generate the hairpin structures respectively. The PCR fragments were inserted into plasmid pFGC5941 with different orientation with the restriction enzyme cutting sites. (b) Real-time RT-PCR of the relative *ZmPIN1a* transcript expression levels in the root. R22 and R31 were the lines of *ZmPIN1a* RNAi-2, r15 and r28 were the lines of *ZmPIN1a* RNAi-3, and WT was the wild type control DH4866. (c) Seedlings of the *ZmPIN1a* RNAi T2 and wild type control DH4866 cultured in nutrient solution. Lines were same to (b). (d) The *ZmPIN1a* UFMu mutants from maize stock center, the triangle represents the insertion. (e) RT-PCR of the relative *ZmPIN1a* transcript expression levels in the root of UFMu mutant mu1005305, mu1040369 and wild type control W22. (f) Seedlings of the *ZmPIN1a* UFMu mutants and wild type control W22 cultured in nutrient solution.

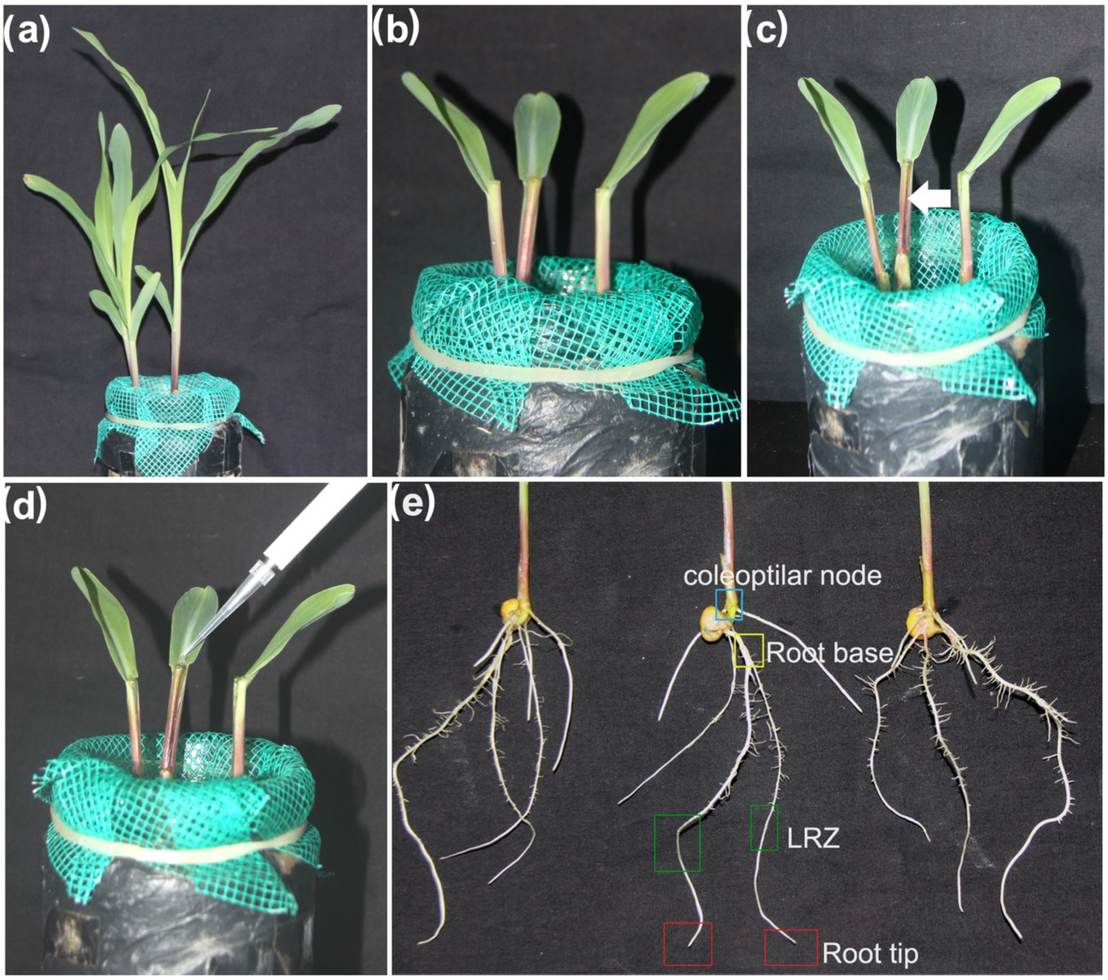

Supplementary figure 3 The protocol of IAA transport capacity determination by using ^{3}H-IAA and IAA, NAA, NPA treatment

(a) Maize plants grow to 2-3 leaves cultured in the nutrient solution. (b) The young leaf was cut off and the first leaf was kept to maintain the photosynthesis. (c) Then the sheaths were carefully nicked vertically by a needle. (d) The hormone solution was dripped to the apex of the maize sheath ((5μl ^{3}H-IAA, 4μl IAA (unlabeled, 100mM) were used) in four times. For the first two, 2.5μl ^{3}H-IAA solution were used respectively, and the last two times 2μl unlabeled IAA solution (100mM) were used respectively. Then the plants were remained to culture for IAA transports determination. (e) The coleoptilar node, root base, LRZ and root tips were carefully removed, cleansed, grinded, weighted and re-suspensioned, and then the disintegrations per minute (d.p.m.) were determined.

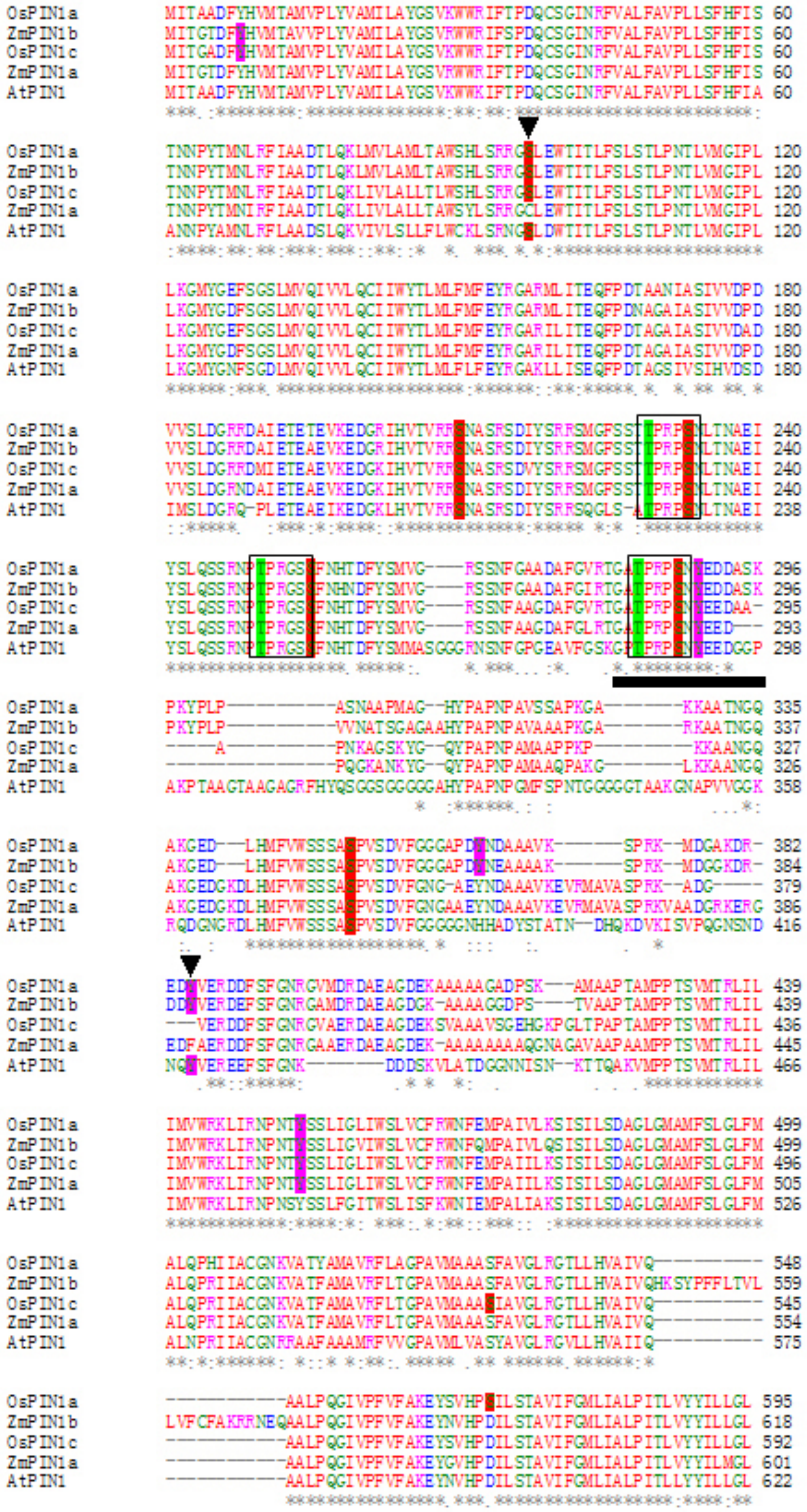

Supplementary figure 4 Sequence alignment and phosphorylation site prediction of ZmPIN1a, ZmPIN1b, OsPIN1a, OsPIN1c and AtPIN1

Background color showed the candidate phosphorylation sites predict by KinasePhos (Huang, Lee, Tzeng & Horng, 2005). The three TPRXS(N/S) motifs (Huang, Zago, Abas, van Marion, Galvan-Ampudia & Offringa, 2010, Michniewicz, Zago, Abas, Weijers, Schweighofer, Meskiene, Heisler, Ohno, Zhang, Huang, Schwab, Weigel, Meyerowitz, Luschnig, Offringa & Friml, 2007) are boxed, which could be reversible phosphorylation by Ser/Thr protein kinase PINOID (PID) and PP2A. The black triangles indicate the missed candidate phosphorylation sites of ZmPIN1a predicted by KinasePhos algorithm compared to OsPIN1a, AtPIN1 and ZmPIN1b. The number of amino acids is indicated on the right. Below the multiple sequence alignment, a black box indicates the oligopeptide targeted by the anti-AtPIN1 antibody (aP20 sc-27163, Santa Cruz Biotechnology).

Reference:

Huang F., Zago M.K., Abas L., van Marion A., Galvan-Ampudia C.S. & Offringa R. (2010) Phosphorylation of Conserved PIN Motifs Directs Arabidopsis PIN1 Polarity and Auxin Transport. *Plant Cell*, **22**, 1129-1142.

Huang H.D., Lee T.Y., Tzeng S.W. & Horng J.T. (2005) KinasePhos: a web tool for identifying protein kinase-specific phosphorylation sites. *Nucleic Acids Research*, **33**, W226-W229.

Michniewicz M., Zago M.K., Abas L., Weijers D., Schweighofer A., Meskiene I., Heisler M.G., Ohno C., Zhang J., Huang F., Schwab R., Weigel D., Meyerowitz E.M., Luschnig C., Offringa R. & Friml J. (2007) Antagonistic regulation of PIN phosphorylation by PP2A and PINOID directs auxin flux. *Cell*, **130**, 1044-1056.

Supplementary Table 1 PCR primers for ZmPIN expression analysis and the identification of transgenic plants

Name	Sense primer	Antisense primer	
ZmPIN1a	ATGATCACCGGCACGGACTT	TCACAGCCCCATCAGGATGTA	
ZmPIN1b	ATGATTACGGGGACGGACTT	TCACAGGCCGAGCAGGAT	Primers used for gene clone
ZmPIN1c	CACCTCGCCCGCCTAAATAA	CAAGCGCCAAACAATGTCGTA	
EPSPS	TGACGCACAATCCCACTATCC	CTTCGCGCTCATTCTCTAAT	Primers used for PCR and probe of Southern blot
ZmPIN1a-SB	CCCTAGCTCAATCCTTGCG	ACGGTGACGTGTATCTTGCC	hybridization
bar	TTGGAGAGGACACGCTGAAATCA	GCTGCCAGAAACCCACGTCAT	Primers used for PCR of the RNAi lines
pin1a-a1		AAGGGTTTGATGCTGAAGGA	Primers used for PCR of the UFMu lines
pin1a-a2		CGTGCGTCGTCAGTAAACAAAAC	compatible with the Mu TIR specific primer
QZmActin1	ATCACCATTGGGTCAGAAAGG	GTGCTGAGAGAAGCCAAAATAGAG	Real-time RT-PCR for reference gene
QZmAUX1	TCTGGTTCCTCGCCATCATCTTC	CGTAAGCGCCTGTCCACCCT	
QZmPIN1a	CGGATAATCGCGTGCGGGAACA	CCGAAGATGACTGCCGTGCTGA	
QZmPIN1b	GCCATGTTCAGTCTCGGGCTGTT	CGGTGCTGAGAATGTCAGGGTGC	
QZmPIN1c	TCATCGCGTGCGGGAACAAG	ACGGCTGTGCTCAGGATGTCG	
QZmPIN3a	GTCGCGTTCCGGTGGCACAT	GGCACGATCCCTTGCGGTAGAG	Real-time RT-PCR for target genes
QZmPIN3b	TCATCTCGTTCCGATTCCACTTCG	TGGTGGCGTGCAGGCTGTACTCT	
QZmPIN5	CCAAGTCGGGCACGGGAATG	CAGACGAGTGGCTGGCGATGAC	
QZmPIN8	TCGCCACGCTCAACAACACGC	GGTTCCTCGCCACCTTCTTCCA	
QZmPIN9	TCGCCATGTACGGCAAGTGGG	CGTCGTCATCAGCGGCTTCCTC	

Supplementary Table 2 Biomass of *ZmPIN1a/b* transgenic lines and wild type control in nutrient solution

	Line	DW_Shoot	DW_Root	DW_Plant	R/S ratio
DH4866	WT	0.22±0.03	0.06±0.011	0.28±0.04	0.26±0.04
ZmPIN1a	A13	0.29±0.02**	0.10±0.01**	0.39±0.02**	0.35±0.04
sense	A17	0.33±0.03**	0.11±0.01**	0.42±0.02**	0.30±0.06
lines	A19	0.27±0.01**	0.09±0.01**	0.36±0.02	0.32±0.04*
	A21	0.27±0.02*	0.08±0.00**	0.35±0.01**	0.30±0.02
	A25	0.26±0.01**	0.09±0.01**	0.35±0.02**	0.34±0.02
ZmPIN1a	a37	0.24±0.02	0.07±0.01*	0.30±0.02	0.29±0.03
antisense	a45	0.23±0.01	0.06±0.01*	0.30±0.01*	0.26±0.06
lines	a55	0.25±0.01**	0.07±0.01**	0.32±0.01**	0.28±0.03*
ZmPIN1b	B19	0.38±0.03**	0.13±0.02**	0.51±0.05**	0.34±0.03**
sense	B23	0.30±0.01**	0.11±0.01**	0.41±0.01**	0.35±0.02**
lines	B25	0.34±0.03**	0.12±0.02**	0.45±0.03**	0.34±0.03**
	B31	0.43±0.02**	0.13±0.02**	0.56±0.02**	0.32±0.06**
	B35	0.36±0.04**	0.12±0.01**	0.48±0.04**	0.34±0.03**
	B37	0.37±0.04**	0.11±0.02**	0.48±0.06**	0.28±0.04
ZmPIN1b	b1	0.19±0.03	0.06±0.01	0.26±0.04	0.34±0.05
antisense	b11	0.25±0.04	0.07±0.01*	0.33±0.04	0.29±0.05
lines	b15	0.18±0.02*	0.05±0.01	0.24±0.03*	0.29±0.06
	b17	0.20±0.03	0.06±0.01	0.25±0.05	0.28±0.02

The prefix “A” or “B” in a line name denotes the *ZmPIN1a* or *ZmPIN1b* overexpression line, an “a” or “b” in a line name denotes the *ZmPIN1a* or *ZmPIN1b* antisense line. WT denotes the wild-type control DH4866. DW was the shorthand of dry weight. The values are the means ± sd. The asterisks indicate significant differences between transgenic lines and WT at the *0.05 or**0.01 level using the *t*-test (*n*=10).

Supplementary Table 3 Root morphology analysis of *ZmPIN1a/b* lines and the wild type control

Lines	Number of roots						Length of roots (cm)				
	Crown roots	Seminal roots	Lateral roots on primary roots	lateral roots on seminal roots	Sum of lateral roots	Total number of roots	Total crown roots	Primary root	Total seminal roots	Total lateral roots	Total length of roots
WT	6.50±1.12	3.25±0.43	68.00±6.36	104.50±12.20	172.50±7.30	183.25±8.58	20.95±6.72	16.90±0.92	39.43±2.47	195.13±12.06	272.40±9.72
A13	6.75±0.83	3.75±0.43	105.5±17.74*	145.00±20.29*	250.50±35.15*	262.00±35.16*	23.84±2.90	19.66±1.97*	55.5±4.81**	215.12±11.5*	314.12±8.41**
A15	7.75±0.83	3.75±0.83	83.00±6.12*	157.50±8.02**	240.50±9.73**	253.00±10.17**	35.00±7.98*	18.68±1.09*	52.25±9.83*	223.52±21.13*	329.44±19.13**
A17	7.25±1.79	3.75±2.05	89.75±6.10**	185.00±18.12**	274.75±18.07**	286.75±18.86**	36.94±10.94*	19.03±1.82*	51.31±5.49**	233.12±17.30**	340.39±23.29**
A19	7.00±0.71	4.00±0.71	86.50±10.59*	138.00±5.10**	224.50±12.89**	236.50±12.18**	20.48±3.92	18.09±1.62	50.89±6.01*	233.28±18.66**	322.74±19.05**
A21	5.50±1.12	3.75±0.43	85.25±7.36**	123.50±12.66*	208.75±6.87**	219.00±7.58**	18.66±4.84	17.26±0.96	51.36±3.95**	226.70±20.00*	313.98±18.30**
A25	8.25±0.43*	2.50±0.50*	131.00±6.44**	127.25±4.82*	258.25±3.90**	270.00±3.67**	23.29±12.74	19.51±1.82*	37.45±1.81	228.12±19.29*	308.38±30.91
a37	6.50±0.50	3.50±0.50	66.50±6.58	96.00±11.64	162.50±10.74	173.50±10.90	24.68±9.23	18.05±1.00	33.35±9.75	185.16±44.03	261.25±28.68
a45	5.75±0.83	2.75±0.43	66.25±10.66	89.75±14.13	156.00±7.65**	165.50±8.02*	18.09±7.00	16.22±0.90	21.97±4.76**	205.45±19.13	261.73±24.54
a55	6.75±0.83	2.50±0.50*	72.75±4.02	94.75±11.08	167.50±12.09	177.75±11.01	23.54±1.08	15.87±2.45	28.74±6.44*	212.17±14.81	280.31±18.10
B19	6.75±1.30	4.00±1.22	79.75±8.55*	128.00±13.66*	207.75±11.08*	219.50±12.46*	27.28±3.52	19.46±1.93*	50.19±2.96**	202.42±10.66*	299.35±11.58**
B23	7.75±0.83	3.00±0	66.00±10.17	124.00±14.25*	190.00±20.06	201.75±20.27	28.78±3.51	14.22±1.15**	39.98±5.96	202.57±30.81	285.54±34.83
B25	7.25±0.83	4.25±0.43*	67.25±11.19	94.75±19.83	162.00±9.27	174.50±10.31	24.76±1.40	15.70±2.92	43.73±3.53	209.50±9.92	293.69±13.98*
B31	7.50±1.66	4.00±1.22	64.75±8.58	127.50±14.22*	192.25±12.44*	204.75±13.79*	28.55±5.76	17.27±4.99	41.95±12.36	231.37±3.96**	319.14±13.92**
B35	6.50±0.87	4.00±1.58	79.25±6.18*	124.00±7.52*	203.25±7.66**	214.75±6.87**	21.47±2.39	15.80±1.89	49.62±10.45	221.54±11.25*	308.42±3.84**
B37	6.75±0.43	5.00±0**	77.50±9.94	133.25±15.99*	210.75±19.78*	223.50±19.78*	24.93±7.38	18.21±1.60	50.44±4.21**	233.30±17.81*	326.88±18.56**
b1	6.00±1.22	2.75±0.43	48.50±10.90*	98.50±6.80	147.00±15.48*	156.75±15.71*	16.18±4.77	14.52±1.59*	33.02±7.84	172.95±28.94	236.67±20.92*
b11	6.25±0.83	2.50±0.87	79.75±7.95*	105.75±10.59	185.50±4.03*	195.25±4.26*	20.65±3.99	13.12±4.13	31.61±6.00*	190.69±38.58	256.07±29.24
b17	7.00±1.58	4.00±0*	61.00±9.30	91.00±8.40	152.00±12.39*	164.00±11.81*	25.80±3.71	13.16±1.44**	35.33±4.30	146.50±17.57**	220.79±15.28**

The prefix “A” or “B” in a line name denotes the *ZmPIN1a* or *ZmPIN1b* overexpression line, an “a” or “b” in a line name denotes the *ZmPIN1a* or *ZmPIN1b* antisense line. WT denotes the wild-type control DH4866. The values are the means ± sd. The asterisks indicate significant differences between transgenic lines and WT at the *0.05 or **0.01 level using the *t*-test (n=10).

Supplementary Table 4 Yields under moderate and high density planting in the fields

	Lines	NO.of ears	Ears/plant	Kernal weight/ear(g)	Yield/plot(kg)	Yield/ha(t)	Yield increasement (%)		
							a	b	c
Moderate density (Dongying, 2011)	WT	48.57±1.69	0.88±0.03	43.83±2.78	2.13±0.18	2.84±0.24	0	-	-
	A13	60.83±2.20**	1.11±0.04**	33.64±2.47**	2.05±0.21	2.73±0.28	-3.87	-	-
	A17	58.83±0.32**	1.07±0.01**	40.16±2.44	2.36±0.13	3.15±0.17	10.92	-	-
	A19	58.80±2.74**	1.07±0.05**	38.42±3.52	2.25±0.10	3.00±0.14	5.63	-	-
	a45	48.01±1.99	0.87±0.04	41.49±2.41	2.00±0.20	2.66±0.26	-6.34	-	-
	a55	47.21±3.86	0.86±0.07	41.15±1.77	1.95±0.23	2.60±0.30	-8.45	-	-
High density (Dongying, 2011)	WT	69.33±2.46	0.77±0.03	35.72±2.02	2.48±0.16	3.30±0.21	0	16.2	16.2
	A13	77.38±1.38**	0.86±0.02**	37.05±1.65	2.87±0.18*	3.83±0.24*	16.06	40.29	34.86
	A17	78.46±2.65**	0.87±0.03**	41.69±1.93**	3.28±0.26**	4.37±0.34**	32.42	38.73	53.87
	A19	80.97±3.09**	0.90±0.03**	37.53±1.50	3.04±0.22**	4.06±0.30**	28.18	35.33	42.96
	a45	73.64±3.34	0.82±0.04	34.72±2.55	2.57±0.30	3.42±0.40	2.73	28.57	20.42
	a55	73.09±1.05	0.81±0.01	33.43±2.86	2.45±0.24	3.26±0.32	-1.21	25.38	14.79
Moderate density(Jinan, 2012)	WT	49.00±2.24	0.89±0.04	41.50±2.15	2.03±0.16	2.71±0.21	0	-	-
	A13	58.75±2.17**	1.07±0.04**	41.18±1.63	2.42±0.08**	3.22±0.10**	18.77	-	-
	A17	56.50±1.12**	1.03±0.02**	42.35±2.46	2.39±0.12**	3.19±0.16**	17.51	-	-
	A19	56.75±2.17**	1.03±0.04**	42.40±1.13	2.41±0.09**	3.21±0.12**	18.20	-	-
	a45	48.75±1.30	0.89±0.02	40.88±1.19	1.99±0.10	2.66±0.14	-2.02	-	-
	a55	47.00±2.74	0.85±0.05	40.00±2.17	1.88±0.07	2.50±0.10	-7.84	-	-
High density(Jinan, 2012)	WT	67.75±1.79	1.23±0.03	33.58±1.76	2.27±0.12	3.03±0.17	0	11.75	11.75
	A13	80.75±3.49**	1.47±0.06**	38.95±1.60**	3.14±0.10**	4.19±0.14**	38.13	29.98	54.37
	A17	83.00±1.87**	1.51±0.03**	39.20±2.42**	3.25±0.18**	4.34±0.24**	43.00	36.00	59.81
	A19	82.25±2.86**	1.50±0.05**	40.43±2.47**	3.32±0.19**	4.43±0.25**	46.09	38.12	63.26
	a45	70.00±2.92	1.27±0.05	34.08±1.69	2.38±0.08	3.18±0.11	4.74	19.47	17.05
	a55	70.50±2.29	1.28±0.04	33.43±1.41	2.36±0.16	3.15±0.22	3.72	25.78	15.92

[a] yield increase of a transgenic line compared with WT under same density cultivation. [b] yield increase of a line in high density than that of the line under moderate density cultivation. [c] yield increase of a transgenic line compared with WT under moderate density cultivation. The values are the means ± sd. The asterisks indicate significant differences between transgenic lines and WT at the *0.05 or**0.01 level using the *t*-test (*n*=5).

Supplementary Table 5 Root number of *ZmPIN1a* lines and WT plants cultured in nutrient solutions with SP or LP

Lines*	Solutions	Crown root	Seminal roots	Lateral roots on crown roots	Lateral roots on primary roots	Lateral roots on seminal roots	Sum of lateral roots	Sum of roots*
WT	SP	2.40±1.50	4.0±2.45	0	88.60±6.65	125.60±12.45	214.20±13.78	221.60±15.29
	LP	3.0±1.67	1.60±0.49	5.80±1.17b	69.20±11.30b	70.80±4.07b	145.80±12.64b	151.40±13.66b
A13	SP	3.80±0.51	3.80±0.40	3.40±2.80a	139.40±9.02a	175.40±21.73a	318.20±20.80a	326.80±20.66a
	LP	4.60±2.05	2.0±0.63b	3.60±0.63ab	136.60±9.58a	144.0±23.15ab	284.20±21.59ab	291.80±20.23ab
A17	SP	3.60±1.02	3.20±0.75	0	166.0±31.81a	210.80±27.26a	376.80±43.61a	384.60±43.66a
	LP	4.20±0.98	4.0±0.63a	10.0±2.45ab	134.20±20.21a	137.20±57.86ab	281.40±56.15ab	290.60±55.28ab
A19	SP	3.20±0.75	3.60±0.80	1.20±2.40	158.40±12.77a	198.20±14.05a	357.80±14.70a	365.60±15.00a
	LP	3.40±1.02	3.80±2.71	2.40±2.54b	135.20±12.69ab	125.0±6.36ab	262.60±29.03ab	270.80±27.27ab
A21	SP	3.80±0.75	3.60±1.74	0.40±0.80	120.80±22.32a	184.0±33.42a	305.20±31.01a	313.60±32.67a
	LP	2.80±0.40b	3.40±1.36a	1.40±2.80a	133.80±7.88a	105.0±43.69b	240.20±42.45ab	247.40±42.92ab
a37	SP	3.40±1.96	2.20±0.98	5.20±5.85	75.60±9.29a	116.60±37.05	197.40±38.66	204.0±38.26
	LP	4.20±1.17	6.20±3.12ab	1.40±1.74a	77.80±6.79	69.0±4.34b	148.20±9.11b	159.60±10.67b
a45	SP	4.40±1.50a	3.2±1.60	0	68.40±7.89a	84.0±20.57a	152.40±19.33a	161.0±19.95a
	LP	3.60±1.02	2.40±0.49a	9.20±12.11	76.20±6.37	69.80±9.22	155.20±11.21	162.20±10.91
a55	SP	3.80±0.98	2±1.41	2.0±2.76	82.20±17.74	85.60±12.64a	169.8±16.03a	176.60±14.79a
	LP	3.0±1.41	2.0±1.10	8.20±12.99	60.80±5.95b	64.20±9.13b	133.20±15.93b	139.20±16.22b

The prefix "A" means the overexpression lines of *ZmPIN1a*, the prefix "a" means the antisense lines of *ZmPIN1a*. WT was shorthand of wild type control DH4866. Values are the means ± sd. The suffix "a" indicates significant differences between a transgenic line and WT in the same nutrient solutions and suffix "b" indicates significant differences between a same genotype in SP and LP nutrient solutions at 0.05 level using the *t*-test (n=10). *: included one primary root.

Supplementary Table 6 Root length of *ZmPIN1a* lines and WT cultured in nutrient solutions

Lines*	Solutions	Total crown roots (cm)	Primary root (cm)	Total seminal roots (cm)	Total lateral roots (cm)	Root system (cm)
WT	SP	4.68±3.42	12.73±1.37	41.25±5.13	290.73±16.31	349.37±12.02
	LP	6.10±1.09	13.0±1.50	19.06±3.95b	229.22±15.35b	267.38±14.23b
A13	SP	11.39±5.49a	27.48±2.08a	57.51±5.66a	248.56±37.63a	344.94±42.27
	LP	39.40±29.28a	28.84±28.84a	43.05±14.27a	269.07±32.89a	380.37±26.63a
A17	SP	10.40±3.63a	26.22±1.45a	65.61±17.28a	298.37±16.07	400.59±20.14a
	LP	26.42±10.55ab	30.99±5.11ab	82.82±23.28a	219.15±10.79ab	359.42±30.10ab
A19	SP	9.66±4.29a	23.79±2.99a	52.19±7.19a	234.86±19.86a	320.49±15.17a
	LP	26.82±10.69ab	28.04±3.36ab	42.18±28.17a	242.09±29.37	339.13±17.12a
A21	SP	8.29±1.64a	27.39±1.31a	45.67±11.27	215.50±28.48a	296.84±24.48a
	LP	10.91±4.39a	28.64±4.56a	65.1±29.16a	159.55±23.07ab	265.00±21.07b
a37	SP	11.36±10.31	12.62±5.86	30.64±1.91a	294.17±45.37	348.79±45.69
	LP	16.67±3.61a	15.38±1.18a	49.96±15.84ab	294.54±29.91a	376.54±25.57a
a45	SP	12.37±3.45a	11.94±2.29	31.93±9.36	271.50±27.62	327.74±28.24
	LP	15.64±7.42a	16.85±2.00ab	27.36±4.66a	298.28±40.32a	358.13±33.80a
a55	SP	13.24±7.07a	13.98±2.31	15.71±10.47a	264.13±21.34a	307.06±17.50a
	LP	13.33±3.97a	13.56±2.88	17.25±10.67	249.17±34.34	293.30±36.28

The prefix “A” means the overexpression line of *ZmPIN1a*, the prefix “a” means the antisense line of *ZmPIN1a*. WT was shorthand of wild type control DH4866. The values are the means ± sd. The suffix “a” indicates a significant difference exists between a line and WT in the same nutrient solution, and suffix “b” indicates a significant difference of a line exists between SP and LP nutrient solutions at 0.05 level using the *t*-test (n=10).

Supplementary Table 7 Determination of 3H-IAA radioactive in *ZmPIN1a* transgenic lines and WT control

Lines	Tissue	^{3}H-IAA Radioactive				Proportion			
		12h	24h	36h	48h	12h	24h	36h	48h
WT	Coleoptilar node	146.08±3.04	109.26±5.80	90.49±5.80	69.71±3.64	0.95±0.00	0.56±0.02	0.45±0.03	0.34±0.02
	Root base	2.16±0.14	39.61±4.20	41.62±8.29	28.25±3.84	0.01±0.00	0.20±0.00	0.21±0.04	0.14±0.02
	LRZ	3.13±0.56	20.77±3.07	40.28±2.88	53.73±4.79	0.02±0.00	0.11±0.01	0.20±0.01	0.26±0.02
	Root tip	2.62±0.30	25.09±3.28	28.45±1.24	55.51±5.75	0.02±0.00	0.13±0.01	0.14±0.01	0.27±0.02
	Total	153.98±2.91	194.73±16.34	200.84±1.62	207.20±3.06	1	1	1	1
A17	Coleoptilar node	152.86±6.52	70.82±8.65**	36.35±0.77**	17.13±1.55**	0.90±0.01**	0.33±0.03**	0.17±0.01**	0.07±0.01**
	Root base	4.06±0.33**	44.83±0.55	42.79±5.44	32.55±4.56	0.02±0.00*	0.21±0.01	0.20±0.04	0.14±0.02
	LRZ	5.87±2.77	58.05±5.70**	75.30±13.37*	101.72±5.54**	0.03±0.01	0.27±0.04**	0.34±0.04*	0.43±0.02**
	Root tip	6.69±0.21**	41.53±4.59**	63.99±3.43**	86.02±11.50*	0.04±0.00**	0.19±0.01**	0.29±0.00**	0.36±0.05
	Total	169.47±8.76	215.24±8.08	218.43±12.14	237.41±0.15**	1	1	1	1
A19	Coleoptilar node	133.96±10.55	65.32±13.16*	35.92±1.84**	19.86±1.45**	0.91±0.00**	0.29±0.04**	0.15±0.02**	0.09±0.01**
	Root base	2.72±0.42	42.84±4.71	43.82±3.38	26.38±1.17	0.02±0.00*	0.19±0.01	0.19±0.01	0.13±0.01
	LRZ	5.68±0.34**	68.43±4.60**	83.91±18.91	82.99±7.99**	0.04±0.01*	0.30±0.04*	0.35±0.04*	0.39±0.04*
	Root tip	5.24±1.33	48.38±3.34**	73.17±4.82**	81.56±7.68*	0.04±0.01*	0.22±0.00**	0.31±0.01**	0.39±0.04*
	Total	147.61±11.97	224.97±16.61	236.82±25.26	210.79±0.58	1	1	1	1
a49	Coleoptilar node	144.50±4.93	114.29±9.51	103.59±11.94	70.66±12.12	0.94±0.01	0.61±0.02*	0.49±0.04	0.35±0.05
	Root base	2.91±0.65	38.92±5.19	41.04±3.48	30.46±3.47	0.02±0.00	0.21±0.04	0.19±0.02	0.15±0.01
	LRZ	2.88±0.63	15.39±1.46	42.41±1.14	48.15±3.72	0.02±0.00	0.08±0.00**	0.20±0.01	0.24±0.03
	Root tip	2.86±1.11	19.12±3.52	26.16±0.45	51.62±3.63	0.02±0.01	0.10±0.01	0.12±0.01*	0.26±0.03
	Total	153.14±3.85	187.72±9.30	213.20±6.87	200.89±8.24	1	1	1	1
a55	Coleoptilar node	150.20±7.51	127.61±12.65	117.07±6.02**	89.19±14.58	0.95±0.01	0.62±0.02**	0.49±0.01	0.41±0.04
	Root base	2.44±0.66	40.54±9.22	44.54±9.72	29.95±5.69	0.02±0.00	0.20±0.02	0.19±0.05	0.14±0.02
	LRZ	3.22±0.84	15.81±2.24	43.18±2.88	46.27±3.99	0.02±0.01	0.08±0.00*	0.18±0.01	0.21±0.03
	Root tip	2.28±0.49	20.23±1.60	32.74±9.35	53.01±0.24	0.01±0.00	0.10±0.00**	0.14±0.03	0.24±0.02

	Total	158.14±6.84	204.18±25.70	237.53±8.54**	218.43±16.04	1	1	1	1
a55*b7	Coleoptilar node	164.97±12.82	133.09±9.42*	112.79±10.08*	94.50±8.45*	0.95±0.01	0.64±0.04	0.54±0.02**	0.44±0.02**
	Root base	3.20±1.10	38.38±7.20	43.96±1.61	30.37±0.82	0.02±0.00	0.19±0.04	0.21±0.00	0.14±0.00
	LRZ	3.09±0.61	14.35±3.84	31.51±0.89*	42.01±3.49*	0.02±0.00	0.07±0.02	0.15±0.01**	0.19±0.02*
	Root tip	2.08±0.75	21.48±3.62	22.54±0.53**	49.95±2.62	0.01±0.00	0.10±0.02	0.11±0.00**	0.23±0.00
	Total	173.34±15.28	207.30±1.99	210.79±11.33	216.82±8.41	1	1	1	1
b1*a49	Coleoptilar node	146.67±10.27	125.57±7.00*	117.90±12.94*	88.14±10.47	0.96±0.02	0.61±0.00*	0.52±0.01*	0.42±0.01*
	Root base	1.94±1.51	43.34±1.29	45.22±2.49	35.17±1.52	0.01±0.01	0.21±0.01	0.20±0.01	0.17±0.02
	LRZ	1.80±0.71	12.61±1.49*	34.68±1.48	35.25±5.56**	0.01±0.01	0.06±0.00**	0.15±0.01*	0.17±0.01**
	Root tip	1.98±1.21	24.16±2.78	29.65±2.84	52.24±6.81	0.01±0.01	0.12±0.01	0.13±0.00	0.25±0.01
	Total	152.40±6.84	205.68±12.56	227.46±19.76	210.79±21.31	1	1	1	1

The IAA transport capacity determination were performed according a procedure in Supplementary Figure 3 by using ^{3}H-IAA, the values in the ^{3}H-IAA Radioactive were the radioactive per mg fresh weight. The values in Proportion were the data of radioactive (per mg fresh weight) of the sample divided by the summation of radioactive of the plant (Coleoptilar node+ Root base+ LRZ+ Root tip). The prefix “A” means the sense line of *ZmPIN1a*, the prefix “a” means the antisense line of *ZmPIN1a*. WT was shorthand of wild type control DH4866. The a55*b7, b1*a49 were the pyramid of *ZmPIN1a* antisense lines and *ZmPIN1b* antisense lines. The values are the means ± sd. The asterisks indicate significant differences between transgenic lines and WT at the *0.05 or **0.01 level using the *t*-test (*n*=3).

Supplemental Table 8 Differentially expressed genes involved in plant hormone metabolism and the signaling process in the root

Gene	A17/WT	Annotation	Homologous in Arabidopsis
GRMZM2G366373	**-1.47**	AUX/IAA32	AT4G14560(IAA1);AT3G23030(IAA2);AT1G04240(IAA3);AT5G43700(IAA4);AT1G15580(IAA5);AT1G52830(IAA6);AT3G15540(IAA19)
GRMZM2G004696	**-1.05**	AUX/IAA7	AT1G80390 (IAA15)
GRMZM2G104176	**-1.01**	ZmIAA4	AT1G51950 (IAA18);AT3G16500 (IAA26);AT5G25890 (IAA28)
GRMZM2G028980	**10.41**	ZmARF16	AT1G30330(ARF6);AT5G37020(ARF8) Class V
GRMZM2G085248	**-1.48**	ZmARF26	AT2G33860(ARF3);AT5G60450(ARF4) Class I
GRMZM2G160005	**-3.95**	ZmARF27	AT5G20730(ARF7);AT1G19220(ARF19) Class VI
GRMZM2G365188	**8.76**	ZmSAUR23	AT3G43120(SAUR39);AT5G20810(SAUR70);AT4G31320(SAUR37) ClassVII
AC196708.3_FGP006	**-2.58**	ZmSAUR46	
GRMZM2G391596	**-1.93**	ZmSAUR55	AT5G53590(SAUR30);AT4G00880(SAUR31);AT2G46690(SAUR32);AT3G61900(SAUR33) ClassXI
GRMZM2G460861	**-2.04**	ZmSAUR4	AT5G20820(SAUR76);AT1G17345(SAUR77);AT1G72430(SAUR78) ClassXVI
GRMZM2G354209	**1.23**	ZmSAUR78	AT1G56150(SAUR71);AT1G79130(SAUR40);AT1G16510(SAUR41);AT3G12830(SAUR72) ClassXIV
GRMZM2G159854	**-8.76**	auxin signaling	AT1G54990 (AXR4)
GRMZM2G041065	**9.33**	H+-translocating inorganic pyrophosphatase	AT1G15690 (AVP1)
GRMZM2G024686	**-2.42**	aspartate kinase	AT1G31230(AK-HSDH_I);AT4G19710(AK-HSDH_II);AT3G02020(AK3); AT5G13280(AK-LYS1);AT5G14060(CARAB-AK-LYS)
GRMZM2G334041	**-9.02**	DNA (cytosine-5-)-methyltransferase	AT1G69770(CMT3);AT1G80740(CMT1);AT3G05430(PWWP domain-containing protein);AT4G08990(DNA (cytosine-5-)-methyltransferase); AT4G13610(MEE57); AT4G14140(DMT2);AT4G19020(CMT2);AT5G27650(PWWP domain-containing protein);AT5G49160(MET1);
GRMZM2G064163	**1.37**	spermidine synthase	AT1G23820(SPDS1);AT1G70310(SPD52);AT5G19530(ACL5); AT5G53120(SPD3);
GRMZM2G135320	**1.17**	S-adenosylmethionine decarboxylase	AT1G69770(CMT3);AT1G80740(CMT1);AT3G05430(PWWP domain-containing protein);AT4G08990(DNA (cytosine-5-)-methyltransferase); AT4G13610(MEE57); AT4G14140(DMT2);AT4G19020(CMT2); AT5G27650(PWWP domain-containing protein);AT5G49160(MET1);
GRMZM2G060369	**8.05**	S-adenosylmethionine decarboxylase	AT3G02470(SAMDC);AT3G25570(S-adenosylmethionine decarboxylase); AT5G15950(S-adenosylmethionine decarboxylase proenzyme 2);AT5G18930(BUD2);
GRMZM2G089856	**-2.91**	aminocyclopropanecarboxylate oxidase	AT1G05010(EFE) AT1G12010 AT1G62380(ACO2) AT1G77330 AT2G19590(ACO1)
GRMZM2G052422	**2.35**		
GRMZM2G126732	**-7.81**		
GRMZM2G039280	**-1.02**	Ethylene-overproduction protein	AT4G02680(EOL1, interacts with and inhibits the activity of ACS5.
GRMZM2G075368	**-4.44**	ETR2 like gene	AT1G04310(ERS2);ERS2(ETR1);AT2G40940(ERS1);AT3G04580(EIN4);AT3G23150(ETR2);
GRMZM2G029323	**-1.37**	EREBP17	AT1G78080 (RAP2.4) related to AP2 4
GRMZM2G003466	**1.48**	EREBP101	AT4G39780 AtERF060
GRMZM2G084264	**-1.92**	EREBP27	AT1G21910 AtERF012
GRMZM2G111415	**7.93**	EREBP15	AT5G25190 AtERF003
AC206951.3_FGP017	**-1.33**	EREBP182	AT1G72360(AtERF073, HRE1)

GRMZM2G369472	**-5.23**	EREBP172	AT3G16770 (ATEBP, EBP, AtERF072, RAP2.3)
GRMZM2G025062	**1.59**	EREBP200	AT3G16770 (ATEBP, EBP, AtERF072, RAP2.3)
GRMZM2G047999	**9.25**	EREBP178	AT1G28160 (ERF087)
GRMZM2G052720	**5.15**	EREBP98	AT3G15210 (ATERF074, ATERF4, ERF4, RAP2.5)
GRMZM2G055180	**-1.21**	EREBP198	AT4G17500 (AtERF1, AtERF100)
GRMZM2G103085	**-7.81**	EREBP139	AT2G33710 (AtERF112)
GRMZM2G100727	**7.82**	EREBP133	AT5G61890 (AtERF114)
GRMZM2G381441	**7.82**	EREBP58	AT3G23240(ERF1)
GRMZM2G022679	**1.77**	gibberellin 3-beta-dioxygenase	AT1G02400(GA2OX6);AT1G30040(GA2OX2);AT1G47990 (GA2OX4);AT1G78440(ATGA2OX1);AT2G34555(ATGA2OX3)
GRMZM2G031724	**2.01**	gibberellin 3-beta-dioxygenase	
GRMZM2G062019	**2.77**	gibberellin receptor GID1L2	AT3G05120(GID1A);AT3G63010(GID1B);AT5G27320(GID1C)
GRMZM2G460383	**1.25**	gibberellin receptor protein	
GRMZM2G440543	**-2.1**	F-box protein GID2	AT4G24210(SLY1)
GRMZM2G023872	**-1.39**	DELLA protein	AT1G14920(GAI);AT1G66350(RGL1);AT2G01570(RGA1); AT3G03450(RGL2);AT5G17490(RGL3)
GRMZM2G017349	**1.34**	phytochrome-interacting factor 3	AT1G09530(PIF3);AT2G43010(PIF4)
GRMZM2G145440	**2.34**	BRI 1-associated receptor kinase 1	-
GRMZM2G015933	**8.03**	BRI 1-associated receptor kinase 1	-
GRMZM2G097258	**8.44**	unknown	AT1G25410(IPT6);AT1G68460(IPT1); AT3G19160(IPT8); AT3G23630(IPT7);AT3G63110(IPT3); AT4G24650(IPT4);AT5G19040(IPT5)
GRMZM2G156127	**-1.38**	cytokinin-N-glucosyltransferase 1	AT5G05860(UGT76C2);AT5G05870(UGT76C1)
GRMZM2G155767	**3.14**	histidine kinase	AT1G27320(AHK3);AT2G01830(AHK4);AT5G35750(AHK2)
GRMZM2G177220	**1.22**	two-component response regulator ARR1	AT3G16857(ARR1);AT4G16110(ARR2);AT4G31920(ARR10);AT1G67710 (ARR11);AT2G25180(ARR12); AT2G01760(ARR14); AT5G58080(ARR18)
GRMZM2G392101	**-2.09**	cytokinin response regulator2	AT1G59940(ARR3);AT2G41310(ARR8);AT3G48100(ARR5);AT1G10470 (ARR4);AT5G62920(ARR6); AT1G19050(ARR7); AT3G57040(ARR9);AT1G74890(ARR15);AT2G40670(ARR16);AT3G56380(ARR17)
GRMZM2G120371	**8.98**	G protein-coupled receptor	AT1G48270(GCR1)

The values were the data of log2 Ratio (A17/WT), and bold type means the difference meets the criteria of log2 Ratio (A17/WT) <-1 or log2 Ratio (A17/WT)>1 and $p<0.001$.

Supplementary Table 9 Promoter analysis of the *ZmPIN1a* and *ZmPIN1b* gene

Element	Function	ZmPIN1a	position	ZmPIN1b	position
MBS	MYB binding site involved in drought-inducibility	AGAAATGTCAATGTGGGG	(-)-243~-249	GATTACTAACTGAATTAT	(+)-470~-476
CCAAT-box	MYBHv1 binding site			CTCTGTCAACGGATGGCA	(+)-1127~-1133
LTR	cis-acting element involved in low-temperature responsiveness			CTACCCGAAATTCCCGAA	(+)-1282~-1288
				TACAATCCGAAACAAACG	(+)-408~-414
				CAACCCAAAGCCCAATGC	(-)-1226~-1232
				AAGCCCAAAGCCCAAGCT	(-)-1259~-1265
				GCCCATAAAGCCCATGGG	(-)-1348~-1354
TC-rich repeats	cis-acting element involved in defense and stress responsiveness	ATATATATATTCTCCAATTACA	(+)-798~-808		
		TAAAGAAACTCTTTTATTTTGG	(-)-693~-703		
HSE	cis-acting element involved in heat stress responsiveness			TCCCGAACAAAATTCGGGTTTC	(+)-1267~-1277
				GCTCAAGCTTATAAGACCCAAC	(-)-1240~-1250
ABRE	cis-acting element involved in the abscisic acid responsiveness	CTCGCCGCCGCGGGGCACCGCG	(+)-81~-91	AAGGTACGTACGTACATATGTA	(+)-640~-650
		GGCTGGCATACGTGCAGAATTT	(+)-267~-277		
		GGGCCGACGTGCCTGCAGCTCC	(-)-45~-55		
		CTGGCATACGTGCAGAAT	(+)-269~-275		
CGTCA-motif	cis-acting regulatory element involved in the MeJA-responsiveness			TTGAAGCGTCAAAGGTA	(+)-656~-661
				CTGACACGTCACTGCCT	(+)-1072~-1077
TGACG-motif	cis-acting regulatory element involved in the MeJA-responsiveness			AACTTCGCAGTTTCCAT	(-)-656~-661
				GACTGTGCAGTGACGGA	(-)-1072~-1077
TCA-element	cis-acting element involved in salicylic acid responsiveness	AGATCGGAGAAGAACACGACAG	(+)-1372~-1382		
O2-site	cis-acting regulatory element involved in zein metabolism regulation			ATTAAGCATGACATGAGTACAT	(+)-1389~-1399
ERE	ethylene-responsive element	TCTCGAATTTCAAATTGTAT	(+)-1047~-1055		
GARE-motif	gibberellin-responsive element	GAAGGAAAACAGAATTGG	(+)-1151~-1157		
		AACTAAAGACAAAATTTAA	(-)-1085~-1092		
P-box	gibberellin-responsive element	TTTAATCCTTTTGGTTAAA	(+)-937~-944		
TGA-element	auxin-responsive element				
CAT-box	cis-acting regulatory element related to meristem expression			CATTATGCCACTTTGGCG	(+)-1005~-1011
				TTCGTTGCCACTTACCCT	(+)-1171~-1177
CCGTCC-box	cis-acting regulatory element related to meristem specific activation			CCAATTCCGTCCGGGCAG	(+)-1051~-1057
GCN4_motif	cis-regulatory element involved in endosperm expression			TCATGTACTGTGTATTTTC	(-)-1377~-1384
Skn-1_motif	cis-acting regulatory element required for endosperm expression	TTTCTAGTCATACAATT	(+)-747~-752	CTCATGTACTGTGTATT	(-)-1380~-1385
		CCAGCTGTCATTTATCC	(+)-315~-320	AATTCGTACTGTACTCA	(-)-1393~-1398

ARE	cis-acting regulatory element essential for the anaerobic induction				
GC-motif	enhancer-like element involved in anoxic specific inducibility			CCTCCGCCCCCGTACAGT	(+)-368~-374
circadian	cis-acting regulatory element involved in circadian control	TTTATCCAAAAAAATCATTGTT	(+)-630~-640	CAATGCCTAAAAAAACGTGTCG	(-)-1210~-1220
		CCCGCACAATATAATCACGAGC	(+)-479~-489		
		ACCCGGCTAAAGAAACAAGGGG	(-)-498~-508		
MRE	MYB binding site involved in light responsiveness	AAATAAAACCTAATTTCCT	(+)-684~-691		
G-Box	cis-acting regulatory element involved in light responsiveness	GACCGTATGCACGTCTTA	(-)-269~-275	GCCTGACACGTCACTGCC	(+)-1073~-1079
		GGGGTGCACGACTAGGAG	(+)-1437~-1443	CGACCTCACGTCTCCACG	(+)-907~-913
		AGAACACGACAGAGTTTG	(+)-1366~-1372	CCAGATCAGCACGAGGTG	(-)-385~-391
		GGGACCCACGTCCAACGA	(+)-1337~-1343		
		CTGGCATACGTGCAGAAT	(+)-269~-275		
Sp1	light responsive element	GCTCCCCCACCCCACTCATCAC	(+)-118~-128	ATACGGCCACCCCCTCCACACC	(+)-506~-516
		ACTCCACCTCCCGCGTTCCTCC	(+)-148~-158	AGTACCCCACCCTACACTACAC	(+)-346~-356
		TACACCCCTCCCCTCCCCCTCG	(+)-229~-239	GGTGGAGGCGGGGGCATG	(-)-371~-377
		GCGGGTCCCACTGGTTGG	(+)-1286~-1292		
		GCACACCCCTCCGACCCAGGCG	(-)-1217~-1227		
		CATAAACCCACCTTTTGATCTT	(-)-1029~-1039		
		TACTCTCCCCCCCCACGTGCTG	(-)-1443~-1453		
Box 4	part of a conserved DNA module involved in light responsiveness	AGAAATATTAATTTAAAC	(+)-876~-882	ATGCAAATTAATGATTAA	(+)-1406~-1412
		ATTTATTAATTGTTTGTT	(+)-830~-836		
		TCAATTATTAATTGTAGA	(+)-727~-733		
Box I	light responsive element	CTCGAATTTCAAATTGTAT	(+)-1047~-1054	AGCCCAAAACTTTCAACGA	(-)-1298~-1305
3-AF1 binding si	light responsive element	TTTTTGAAGAAATATTTATAAAT	(+)-588~-599		
Box-W1	fungal elicitor responsive element	GTAATTTTGACCCCAAAT	(+)-412~-418		
GT1-motif	light responsive element	CCTTTTGGTTAAATAACA	(+)-932~-938		
		AAACTGAATTGGAATAAA	(-)-773~-779		
		TCCTAGGGTTAAATATAA	(+)-670~-676		
A-box	cis-acting regulatory element			CCAATTCCGTCCGGGCAG	(+)-1051~-1057
ATC-motif	part of a conserved DNA module involved in light responsiveness			ACGAAAAGTAATCTGCTGCT	(+)-877~-885
I-box	part of a light responsive element			AATTTTTATTAGCTAGAATAATT	(+)-431~-442

(+) meas the positive chain,（-）meas the negative chain.
