# Supplementary material for: Enhancing auxin accumulation in maize root tips improves root growth and dwarfs plant height
Source: Plant Biotechnol J. 2017 Jun 23;16(1):86–99. doi: 10.1111/pbi.12751 (PMC5785362; doi:10.1111/pbi.12751)
Supplement: Supplementary file 2 — Data S1 Supplementary Experimental protocol. [file PBI-16-86-s001.docx]

**Supplementary Experimental protocol**

**Measuring plant morphology and biomass**

Hydroponic cultures and LP treatment were performed as described by Li *et al.* (Li et al., 2011). The plant roots were scanned with a scanner (Powerlook 1000, China) and analyzed by using LA-S-type plant image analysis software (developed by Zhejiang University, China). The numbers of axial and lateral roots were counted, and the total root lengths were measured. The roots and shoots were dried in an oven at 80°C to a constant weight and then weighed.

To determine the morphology and biomass of plants in vermiculite and pots, the transgenic and WT lines were grown from May to July under natural conditions. The plants in vermiculite were harvested at 5-leaf stage for morphology and biomass analysis, and the plants in pots were thinned at 3-leaf stage to ensure one plant per pot and then grown to mature stage.

**Cloning *Z. mays* PIN1 genes and sequence analyses**

Total RNA was extracted from maize inbred line DH4866. Maize cDNA was synthesized by following the gene cloning instructions for the SMART cDNA Synthesis Kit. Gene-specific primers were designed as the submitted sequences (Table S1). The amplified cDNA fragment was inserted into a pGEM T-easy vector (Promega) and sequenced. Sequence analysis (Figure S1) was performed with Clustal W2 and MEGA 5 (Tamura et al., 2011). Phosphorylation site prediction was performed with KinasePhos (Huang et al., 2005). Promoter searches for cis-elements were performed using the PlantCARE website (Lescot et al., 2002).

The sense or antisense coding sequences of *ZmPIN1a* or *ZmPIN1b* were inserted in the mini-Ti plasmid pCAMBIA1300-Ppht1:: MCS- P35S::*EPSP* for maize genetic transformation. Both sense or antisense coding sequence of *ZmPIN1a* or *ZmPIN1b* gene was driven by the barley *Pht1* promoter (Schunmann et al., 2004) and the *5-enolpyruvylshikimate-3-phosphate* (*EPSP*) *synthase* gene as a selectable marker gene driven by CaMV35S promoter (Figure 2). For the *ZmPIN1a* RNAi lines, as described in figure S2, in *ZmPIN1a* RNAi-2 the PCR fragment of +930bp~+1287bp was used, and in *ZmPIN1a* RNAi-3 the fragment of +951bp~+1412bp were used to generate the hairpin structures respectively. The PCR fragments were inserted into plasmid pFGC5941 with different orientation with the restriction enzyme cutting sites. Both the gene fragment and selected marker gene *bar* were driven by CaMV35S promoter (P35S).

**Producing and identifying the transgenic lines**

*Agrobacterium*-mediated maize shoot-tip genetic transformation was performed as described by a Chinese patent (CN 1305005A) (Zhang *et al*., 2001). In addition to herbicide screening for the selection marker *EPSP* or *bar*, a PCR assay, Southern blotting and genetic analyses were conducted on the plants at every generation (Figure 2). Genomic DNA extraction, PCR assay and Southern blotting, were performed as described by Li *et al.* (Li et al., 2011). For Western blotting, an anti-AtPIN1 antibody (aP20 sc-27163, Santa Cruz Biotechnology) was used and performed using standard procedures of the kit (WB7108, Invitrogen). Plasma membranes were isolated according to the method of [Abas](http://www.ncbi.nlm.nih.gov/pubmed?term=Abas%20L%5BAuthor%5D&cauthor=true&cauthor_uid=20193653) *et al.* (Abas and Luschnig, 2010) except for the components of the extraction solution (100 mM Tricine/Tris, pH 8.0, 300 mM mannitol, 3 mM MgSO4, 3 mM EGTA, and 0.5% (w/v) polyvinylpolypyrrolidone, 5 mM dithiothreitol (DTT) and 1 mM phenylmethylsulphonyl fluoride (PMSF)). The mitochondrial fraction was the precipitate of the supernatant using 21,000g centrifugation.

Transient expression assays were performed on onion epidermal cells by introducing a plasmid contained Ppht1::*ZmPIN1a* -*GFP* by the particle bombardment method. The bombarded materials were maintained at 22 °C in the dark for 3 days and then were sampled for the GFP fluorescence assay. Images were taken with an Olympus microscope BX51 and an Olympus C-5050ZOOM camera.

**Real-time RT-PCR of candidate genes**

Maize total RNA was extracted from the samples using TRIzol reagent and then treated with RNase-free DNase. cDNA synthesis was performed with an RT reagent kit (TAKARA, China) according to the manufacturer’s protocol. Real-time RT-PCR was performed in an ABI7300 with a SYBR Green RT-PCR Kit (Takara, China), and the reaction volumes and amplification conditions were performed according to the manufacturer’s protocol. The gene transcript levels were calculated using the 2^-ΔΔCt^ method (Livak and Schmittgen, 2001) except given special footnote, and maize *Actin1* (NM_001155179.1) was used as an internal control. The entire experiment was repeated three times, and the primer sequences used in this study are shown in Table S1.

**Determining the IAA concentration and IAA transport capacity determination**

The leaves, stems, LPRs and root tips of *ZmPIN1a* transgenic and WT lines cultured in nutrient solution for 8 days (2-leaf stage) were used for IAA concentration determination. Samples were prepared according to the method of Edlund *et al.* (Edlund et al., 1995). The analysis was performed using a gas chromatograph-mass spectrometer (GC-TOF MS; Agilent/LECO) fitted with a capillary column (DA-35 ms; 30 m 3 0.25 mm^3^ 0.25 mm; Agilent). Hydroponic cultures were performed as described previous.

For IAA transport capacity determination, the young leaves of maize plants at the 2-leaf stage were carefully cut off, and the first leaf was kept to maintain the photosynthesis. Then the sheaths were finely nicked vertically as shown in Figure S3, and the IAA solution was slowly dripped onto the shoot apex. For the first two, 2.5 μl ^3^H-IAA solution were used respectively, and the last two times 2 μl unlabeled IAA solution (100 mM) were used respectively. After infiltration, the plants were cultured for IAA transportation. At the ^3^H-IAA solution feeding for 12, 24, 36 and 48h the maize coleoptilar node, root base, LRZ and root tips were sampled for the ^3^H-IAA determination. The samples were grind by small electric drill respectively and the fresh weights were recorded. And then the homogenate was transferred into 5 ml scintillation fluid to count the radioactivity (disintegrations per minute (d.p.m.)) with a scintillation counter for 1 min. And then the ^3^H-IAA contents in different part of a plant were calculated based on fresh weight. Three independent biological repeats were performed with one sample consist of 4 maize seedlings. For the IAA, NAA and NPA treatment, maize plants were done according to the protocol of IAA transport capacity determination (Figure S3) except for the solutions used. The plants were harvest after 3 days re-cultured for root morphology analysis.

**Yield determination of density gradient experiments**

A density gradient experiment was performed in the experimental fields of Jinan (drought and density gradient, 117°29′E, 36°54′N) and Dongying (density gradient, 118°49′E, 37°46′N) in maize growing season. The trial plots were arranged in a randomized complete block design with four replications. Seeds of each homozygous transgenic and WT lines were sown in five rows for each plot in May with four seeds per hole, and the plants were thinned at the 3-leaf stage to ensure the scheduled densities. The plot was 2.5 m in length with a width of 0.6 m between rows, and the plants in each row were at an interval of 22.5 cm (moderate density, 73,370 plants/ha) or 14 cm (high density, 120,000 plants/ha). Mature ears were harvested to determine their yield (dry weight). The ear length and number of kernel rows were recorded from five randomly selected ears in each plot. The experiment was repeated over two years.

**Drought treatment and yield calculation**

Drought treatment plants in soil pots at the 3-leaf stage grown under conditions of 32°C/25°C (day/night) at a photon flux density of 700 μmol m^-2^ s^-1^ (14 h light/10 h dark) were denied water and rain was avoided. When most of the plants were seriously wilting they were watered well to recover. The plant parameters were recorded at days 2 and 5 of the drought treatment and 2 days after the recovery.

A field experiment was performed in the experimental field in Jinan (117°29′E, 36°54′N) under a rain shelter that was rolled up on sunny days. The trial plots were arranged in a randomized complete block design with three replications. Forty seeds of each homozygous transgenic and WT line were sown in a double row plot in May. The plot was 2.5 m in length with a width of 0.6 m between rows, and the plants in each row were at an interval of 25 cm (66,700 plants/ha). Plants were thinned at the 3-leaf stage to ensure the scheduled densities. At the 10-leaf stage, the plants were subjected to drought stress for six weeks, with the water content of soil maintained from 15%-17% at a depth of 40 cm during the treatment. Then, the plants were watered well. Mature ears were harvested to determine kernel yield, ear length and number of kernel rows.
